# Effectiveness of a teletherapy-based phonological short-term memory training in reducing phonological impairments in the logopenic variant of primary progressive aphasia: a multiple case study

**DOI:** 10.3389/fnhum.2025.1724345

**Published:** 2025-12-18

**Authors:** Guillaume Duboisdindien, Monica Lavoie, Robert Laforce, Joel Macoir

**Affiliations:** ^1^Chaire de Recherche sur les Aphasies Primaires Progressives – Fondation de la Famille Lemaire, Québec City, QC, Canada; ^2^Centre de Recherche en Neurosciences, CHU de Québec, Québec City, QC, Canada; ^3^Université Laval, Québec City, QC, Canada; 4 Clinique Interdisciplinaire de Mémoire (CIME), CHU de Québec, Université Laval, Québec, QC, Canada; 5 Université Laval, Faculté de Médecine, Québec, QC, Canada; 6 Centre de Recherche CERVO, Québec, QC, Canada

**Keywords:** primary progressive aphasia, logopenic variant, phonological short-term memory, teletherapy, repetition training, rehabilitation

## Abstract

The logopenic variant of Primary Progressive Aphasia (lvPPA) is marked by phonological short-term memory deficits that compromise repetition and communication. While previous interventions in PPA have primarily targeted lexical-semantic abilities, little is known about therapies that directly address phonological impairments, primarily through teletherapy. This first study investigated the efficacy of an intensive phonological short-term memory training program delivered via teletherapy in individuals with the lvPPA. The intervention aimed to improve repetition of trained items, promote generalization to untrained items, facilitate transfer to functional tasks, and ensure maintenance over time. In the present study, significant improvements were observed in both immediate and delayed repetition of trained items, with partial short-term generalization to untrained items, particularly for words in delayed tasks. No substantial generalization effects were observed for functional language tasks, including picture description and picture naming, suggesting that the intervention’s impact may remain task specific. Individual trajectories revealed heterogeneous responses, potentially influenced by baseline cognitive profiles, spontaneous strategies, or fatigue. Mixed-effects models confirmed that interindividual factors explained a substantial portion of the variance. These findings support the feasibility and clinical relevance of remote phonological training in the lvPPA and underline the importance of early, personalized interventions. The study also raises the hypothesis that delayed repetition may facilitate internal rehearsal, enhancing generalization. Further research is needed to assess broader functional outcomes and optimize protocol scalability.

## 1 Introduction

Primary progressive aphasia (PPA) represents a relatively rare cause of major neurocognitive disorder, a condition defined in the DSM-5 as a decline in one or more cognitive domains that interferes with functional independence, and typically presents with an insidious onset between 60 and 70 years of age (Neary et al., 1998; Gorno-Tempini et al., 2011). PPA is primarily charaterized by a gradual decline in language abilities, while other cognitive functions remain relatively preserved during the early stages of the disease (Mesulam et al., 2014). Based on neuroanatomical, motor, linguistic, cognitive, and behavioral criteria, three clinical variants of PPA have been described (Gorno-Tempini et al., 2011): the nonfluent/agrammatic variant, the semantic variant, and the logopenic variant (lvPPA). Language and communication impairments are prominent across all three variants; however, this study focuses explicitly on the lvPPA.

Clinically, lvPPA is characterized by marked anomia and impaired sentence repetition (Gorno-Tempini et al., 2011). Diagnosis also relies on the presence of at least three of the following criteria: production of phonological errors, preserved semantic memory, intact articulation and prosody, and absence of clear agrammatism. Phonological errors can hinder speech intelligibility, potentially leading to communication breakdowns, disrupted conversations, and, in some cases, frustration or social withdrawal (Davies et al., 2025). Difficulties with repetition and phonological errors are thought to result, in large part, from deficits in phonological short-term memory (pSTM) (Meyer et al., 2015), which supports the brief maintenance of sound-based information, and, to a lesser extent (see Baddeley, 1992; Baddeley et al., 1998; Majerus, 2013), from working memory impairments, which also recruit executive and attentional control mechanisms (Whitwell et al., 2015).

In lvPPA, brain atrophy is typically observed in the left perisylvian or posterior parietal regions (Gorno-Tempini et al., 2011). However, more recent work highlights considerable variability in the spatial distribution and magnitude of atrophy even in early stages, with involvement extending into superior parietal, anterior temporal, and inferior frontal areas (Conca et al., 2022; Taylor et al., 2025). Mandelli et al. (2023) identified two distinct neural networks involved in auditory-verbal short-term memory and lexical retrieval, anchored in the anterior angular gyrus and the posterior superior temporal gyrus, respectively. These partially dissociable pathways may account for the heterogeneity of the clinical presentation.

These characteristics raise important questions about potential therapeutic targets, particularly in relation to phonological processing and memory systems. Behavioral speech-language therapy remains the primary approach for supporting individuals with PPA. Therapy aims to slow the progression of language decline, prevent social isolation, and provide caregivers with appropriate guidance (Tippett et al., 2015; Cadório et al., 2017).

Telerehabilitation is increasingly recognized as a viable modality for delivering treatment to individuals with neurodegenerative conditions such as PPA. Its clinical feasibility has been demonstrated in both PPA and related populations (Macoir et al., 2017; Dial et al., 2019), offering an accessible alternative for individuals facing mobility challenges or geographic barriers. However, it may also shift some practical demands onto caregivers, who are often required to assist with technology setup, ongoing troubleshooting, and real time session support.

Although several treatment studies, not limited to telerehabilitation, have included individuals with lvPPA, relatively few have explicitly focused on this variant (Newhart et al., 2009; Pagnoni et al., 2021). Existing studies have primarily targeted two domains: cognitive interventions aimed at attention and executive functions that contribute to verbal storage (Sohlberg et al., 2000), and lexical–semantic approaches designed to improve lexical access (Lavoie et al., 2020; Pagnoni et al., 2021). Several studies have also explored phonological interventions in lvPPA, aiming to strengthen underlying phonological representations or to improve speech production through neuromodulation or integrated behavioral approaches (Trebbastoni et al., 2013; Tsapkini and Hillis, 2013; Bereau et al., 2016; Nickels et al., 2025). However, none of these interventions directly targeted the pSTM mechanism itself, despite pSTM being considered the core deficit underlying repetition and phonological impairments in lvPPA. To our knowledge, the present study is the first to implement and evaluate a treatment designed explicitly to strengthen pSTM in lvPPA. While impairment-based interventions may appear reductionist, they play a crucial role in lvPPA because the targeted mechanism, pSTM, constitutes a core computational bottleneck underlying phonological encoding and repetition. Strengthening this process is therefore a theoretically grounded way to support the integrity and accessibility of phonological representations, which are central to multiple language functions.

Treatment principles from post-stroke aphasia, including interventions aimed at verbal short-term memory, are frequently adapted for progressive conditions. In this context, several phonologically focused interventions developed for individuals with conduction aphasia may offer useful models. These interventions are based on the idea that impaired short-term maintenance of phonological representations contributes to repetition and naming deficits (Martin and Saffran, 1997; Majerus et al., 2005). Although generalization is typically more constrained in progressive disorders than in post-stroke aphasia, process-based training may still yield functional benefits when the trained mechanism subserves multiple tasks and remains partially preserved. Our protocol was therefore designed to reinforce residual pSTM capacity, prioritizing the mechanism most affected in lvPPA, rather than relying on lexical–semantic systems, which are relatively preserved in this variant. Many draw on cognitive frameworks such as the interactive activation model (Martin et al., 1996), which attributes repetition deficits to rapid decay of phonological activation.

The aims of pSTM-oriented therapy are to: (a) extend the duration of phonological representations, (b) improve their stability and accessibility, (c) reduce phonological errors, and (d) facilitate transfer to other language tasks (e.g., naming, sentence comprehension, spontaneous speech). Evidence from post-stroke aphasia suggests that directly enhancing pSTM may lead to improved repetition and, in some cases, generalization to broader language functions such as lexical access and sentence-level production (Majerus et al., 2005; Kalinyak-Fliszar et al., 2011; Berthier et al., 2014; Zakariás et al., 2019; Banco et al., 2024). In lvPPA, improved pSTM may plausibly support everyday communication by stabilizing phonological sequences during word retrieval, increasing resistance to phonological decay in conversational contexts, and reducing error cascades that disrupt message formulation. Transfer is expected primarily for tasks that rely on phonological buffering (e.g., naming, sentence repetition), but not for functions that depend on semantic or syntactic systems undergoing progressive decline.

Importantly, few if any studies have examined whether similar gains can be achieved in individuals with progressive aphasia, and particularly in remote delivery formats such as teletherapy.

The purpose of this study was to evaluate the feasibility and efficacy of a teletherapy-based pSTM intervention for individuals with lvPPA. We examined whether the intervention improved repetition accuracy for trained words and pseudowords and whether these gains were maintained at 4 weeks and 3 months post-treatment. We also investigated whether improvements generalized to untrained stimuli and persisted over time, as well as whether the intervention reduced phonological errors in functional language tasks such as picture naming and connected speech, with effects sustained longitudinally.

## 2 Materials and methods

### 2.1 Participants

This study was approved by the Human Research Ethics Committee of the Center Hospitalier Universitaire de Québec, affiliated with Université Laval (HREC reference number: 2024–6872). Informed consent, as approved by the HREC, was obtained from all participants in the presence of a trusted caregiver, in compliance with Canadian national and international ethical standards.

Five participants with lvPPA, fulfilling the diagnostic criteria of Gorno-Tempini et al. (2011), were recruited from the Clinique Interdisciplinaire de Mémoire du CHU de Québec and referred to the research team. All participants had a biomarker-confirmed diagnosis, with three showing positive cerebrospinal fluid profiles and two presenting amyloid-PET positivity. Sociodemographic characteristics are presented in Table 1.

**TABLE 1:** Participants’ sociodemographic characteristics.

Participants	LM01	JRM01	RC01	CG01	ST01
Age	68	55	68	68	54
Gender	F	M	F	F	F
Education (years)	16	16	11	12	11
Socioprofessional category (NOC)	Administrative support	Senior management in information technology	Administrative support	Occupations in art, culture, recreation and sport	Administrative support
Diagnosis date	May 2023	Jan 2023	Aug 2023	Apr 2023	Nov 2023
Time post-diagnosis at T1 (months)	3	7	2	6	2
Positive biomarkers	PET	CSF	CSF	PET	CSF
Pharmaceutical treatment	CI	CI	CI	CI	CI

CI, Cholinesterase inhibitor; CSF, cerebrospinal fluid; PET, Positron emission tomography imaging.

Inclusion criteria required participants to have adequate reading ability, operationally defined as the ability to accurately read aloud short sentences and single words at a functional level during the pre-treatment screening assessment, corrected vision or hearing in cases of presbyopia or presbycusis, the ability to immediately repeat words of at least two syllables, sufficient internet connectivity, the ability to provide informed consent, and long-term use of French as their primary language.

Exclusion criteria included the presence of a comorbid addictive disorder involving alcohol and/or drug use, as defined by DSM-5 criteria, the presence of a comorbid somatic condition known to impact cognitive abilities or brain structures (i.e., expansive cerebral processes, multiple sclerosis, lupus, epilepsy, neurodegenerative diseases other than lvPPA, normal pressure hydrocephalus, and ischemic or hemorrhagic strokes with language-related sequelae), or current participation in speech therapy sessions targeting short-term working memory.

Before the study began, all participants underwent a comprehensive neuropsychological and language assessment to confirm eligibility based on the inclusion and exclusion criteria and to establish their cognitive profiles (Table 2). Baseline cognitive-linguistic performance varied considerably across participants (Table 2). Some individuals showed preserved global cognition, processing speed, and lexical-semantic skills. In contrast, others presented marked impairments in these domains, including reduced MoCA scores, slowed processing speed, and phonological discrimination difficulties. Despite this heterogeneity, all participants met the inclusion criteria and demonstrated sufficient reading, comprehension, and basic repetition abilities to engage meaningfully in the treatment. This variability is clinically relevant, as several of these baseline measures are referenced later in the Discussion to contextualize interindividual differences in treatment responsiveness.

**TABLE 2:** Participant’s summary of neuropsychological and language assessments.

Participants	LM01	JRM01	RC01	CG01	ST01
**Neuropsychological tests**					
**Global cognitive**					
- MoCA (/30)	28	18*	16*	10*	10*
**Processing speed—WAIS IV**					
- DSST (/135)	52	13*	38	0*	27
**Digit span–WAIS IV**					
- Forward span	8	7	7	9	4*
- Backward span	8	4	6	4	5
**Visual memory—DMS 48**					
- Immediate recognition (/48)	48	45	48	48	44
- Delayed recognition (/48)	48	48	39	46	44
**Language tests**					
**Recognition of spoken words—BECLA**					
- Auditory discrimination (/36)	31	35	36	36	36
- Auditory lexical decision (/20)	17*	20	19*	19*	19*
Semantic processing—BECLA					
- Semantic association of pictures (/20)	19	20	19	17*	19
**Spoken production**					
- Spoken word-picture matching BECLA (/20)	20	20	20	20	18*
- Picture naming TDQ-60 (/60)	56*	57*	52*	40*	59
- Picture naming BECLA (/20)	17*	16*	17*	14*	13*
- Rhyme judgment on pictures BECLA (/10)	10	8	7*	0*	7*
**Communication scale—ECVB**					
- Global score (/76)	71.5	60	49	56	41.5
- Expressing intentions (/9)	9	9	9	9	9
- Conversation (/21)	19	18	15	17	12
- Phone call (/21)	20	14	8	10	7
- Social relations (/15)	15	13	9	15	10
- Communication satisfaction skill (/10)	8.5	6	8	5	6.5

*Indicates a score below the norm (≤2 SD) or ≤ 5 th percentile; DMS-48: Visual recognition memory test (Barbeau et al., 2004); Digit Symbol Substitution Test (Wechsler, 1997); WAIS-IV, Wechsler Adult Intelligence Scale (Wechsler, 2008); BECLA, Batterie d’Évaluation Cognitive du Langage chez l’Adulte (Macoir et al., 2016); ECVB, Échelle de Communication Verbale de Bordeaux (Darrigrand and Mazaux, 2000); MoCA, Montreal Cognitive Assessment (Nasreddine et al., 2005); TDQ-60, Test de dénomination de Québec (Macoir et al., 2018).

### 2.2 Study design

**FIGURE 1:**
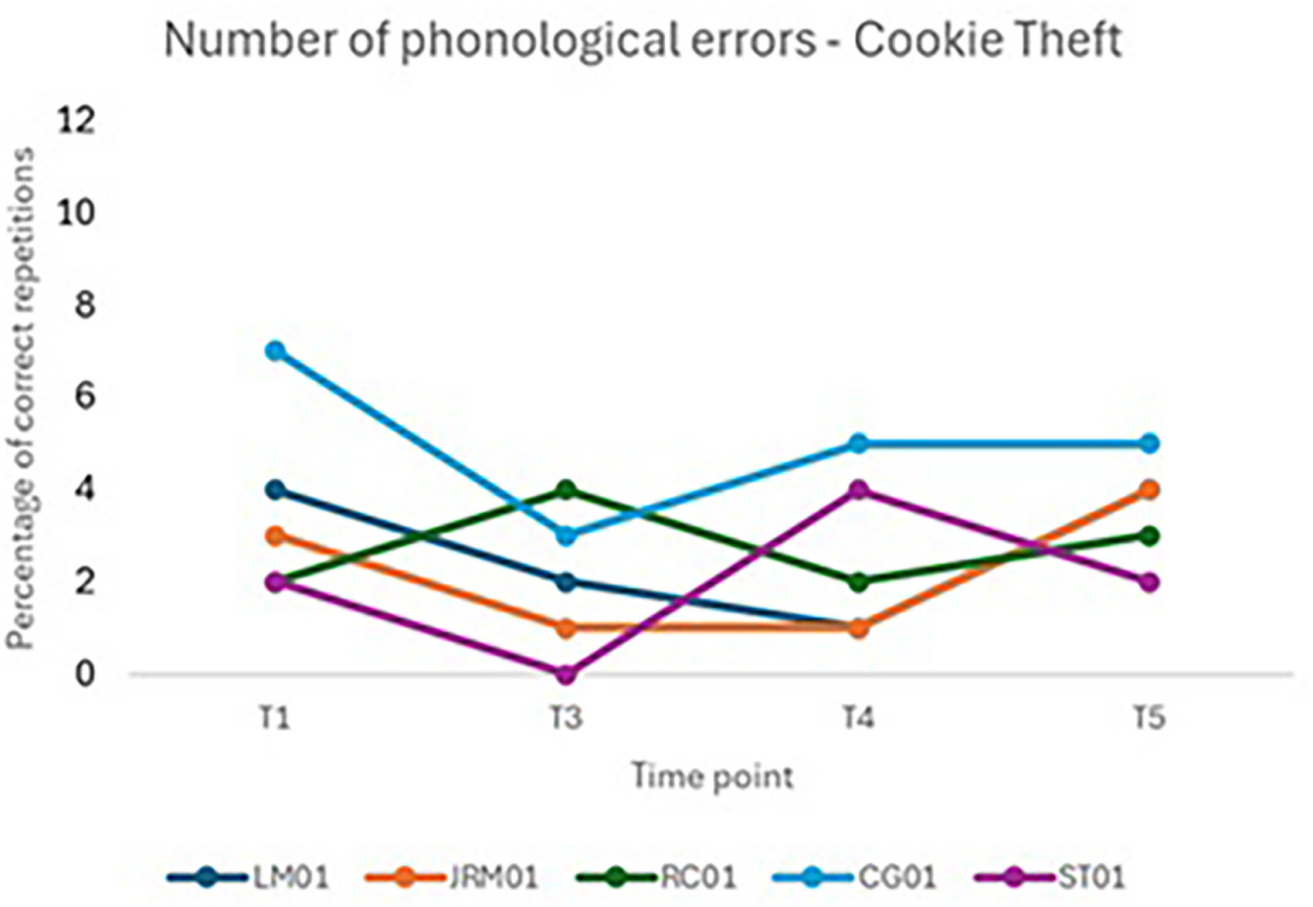
Overview of the assessment timeline (Cookie Theft; Goodglass and Kaplan, 1972; TDQ-60, Test de dénomination de Québec; Macoir et al., 2018).

This study involved three stages: (1) baselines measures, (2) phonological short-term memory training and (3) efficacy, follow-up, and generalization measures (Figure 1).

### 2.3 Materials

#### 2.3.1 Trained and untrained word and pseudoword stimuli (lists A and B)

The speech-language intervention used 400 words and 400 pseudowords, systematically organized by syllabic length (2–8 syllables). Words were selected from the Lexique 3.83 database based on moderate frequency (between 0.5 and 2.99/140,000 words), syllabic length, and syllabic structure, with 80% featuring simple syllables (e.g., CV, CVC). Pseudowords matched the same structural criteria but excluded lexical frequency and were validated for pronounceability by native French speakers. Items at each syllabic level were divided into matched trained and untrained lists based on lexical and phonological properties. Stimuli were presented in pseudorandomized sequences using a PowerPoint macro to ensure balanced difficulty across sessions. Full details are available in Supplementary material. A binary scoring system was applied, with *1* indicating a correct production of the target item and *0* indicating the presence of a phonological error.

#### 2.3.2 Picture naming

The *Test de Dénomination de Québec-60 items* (TDQ-60) (Macoir et al., 2018) is a standardized picture-naming task developed to assess lexical access in adults. It comprises 60 color illustrations depicting concrete nouns of varying lexical frequencies, balanced for phonological and semantic complexity. In this study, particular attention was given to the identification of phonological errors, which were categorized using criteria established in the psycholinguistic and clinical aphasiology literature (Henry et al., 2016; Martin and Saffran, 2002; Nickels and Howard, 1995). Phonological errors included phoneme substitutions, omissions, insertions, metatheses, assimilations, and cluster simplifications. Two trained raters independently coded the responses.

#### 2.3.3 Connected speech

Connected speech was elicited using the *Cookie Theft* picture description task from the Boston Diagnostic Aphasia Examination (Kaplan et al., 1983), which prompts spontaneous narrative discourse in a semi-structured, naturalistic setting. Speech samples were audio-recorded, transcribed verbatim in PRAAT using CorPAGEst conventions (Bolly et al., 2017), and segmented into utterances. Phonological errors were identified based on established criteria and independently coded by two trained raters. Discrepancies were resolved by consensus to ensure interrater reliability.

#### 2.3.4 Satisfaction questionnaire

The post-treatment satisfaction questionnaire comprised 11 items assessing multiple dimensions of patient experience, including perceived benefit, clarity and acceptability of tasks, comfort with technology, and fatigue associated with remote therapy. To enhance comprehension, a simplified 5-point Likert scale (0–4) was employed, ranging from “strongly disagree” to “strongly agree.” Questions also directly addressed the teletherapy modality, focusing on participants’ comfort with computer-based interactions, the perceived usefulness of remote therapist guidance, and the tolerability of session rhythm and duration.

### 2.4 Intervention

The intervention aimed to strengthen pSTM through a structured oral repetition training program delivered over 5 weeks (15 sessions; 3 sessions/week, 45–50 min each). Sessions consisted of four repetition tasks divided into two phases: immediate and delayed repetition. Stimuli were presented live via a secure videoconferencing platform, with PowerPoint macros enabling automatic randomization. During delayed repetition, a 5-s visual countdown and auditory cue signaled the end of the retention interval. To sustain attention and reduce cognitive load, short oral reading passages were interspersed between tasks, using content unrelated to the target stimuli, followed by open conversation. Although oral reading can tax pSTM in lvPPA, it was selected here because reading connected text supports comprehension through semantic and syntactic scaffolding, thereby reducing the demands on phonological buffering relative to isolated word or pseudoword repetition. It also provides a familiar, structured activity that helps regulate cognitive fatigue during intensive repetition practice.

Stimuli (words and pseudowords) were organized by increasing syllabic complexity (2–8 syllables) and adapted to each participant’s proximal zone of development. Baseline performance was determined individually based on pre-treatment assessments (T1–T2). If a participant achieved ≥ 80% accuracy at one level but not at the next, that level was set as their starting point. Each training program included four levels: the baseline level and three progressively more complex levels. When participants were unable to repeat a word correctly, a scaffolding hierarchy of cues was applied (see Table 3).

**TABLE 3:** Scaffolding hierarchy for repetition tasks.

Level	Name	Description
1	Simple Syllabification	The administrator segmented the word orally into syllables and asked the participant to repeat it in standard form. Repetition in syllabified form prompted a second attempt.
2	Syllabification with Visual Support	The syllabified model was repeated with a visual cue (one colored sphere per syllable). The visual was removed before prompting normal repetition.
3	Sustained visual support	The visual cue remained on screen. The administrator guided the participant through each syllable with a pointer, then prompted repetition of the full word.

If a participant was unable to produce the target word after Level 3, the attempt was discontinued, and they were reassured and encouraged to continue. Signs of frustration triggered short breaks and normalization of effort expectations. Repeated failure at a given level, defined as < 80% accuracy on both trained and untrained items over one intervention week and a follow-up session, led to reassignment to the previous level to consolidate progress and prevent overload. Conversely, progression to a higher level was determined when participants consistently achieved an accuracy rate of ≥ 80% on both trained and untrained items across one intervention week and a follow-up session.

Each session was led by a trained speech-language pathologist, recorded (with consent), and documented using individualized Excel files and qualitative logs. Protocol adherence was ensured through standardized instructions, stimulus validation, systematic randomization, and ongoing supervision. Positive reinforcement was provided throughout without additional exposure to training items. Further procedural details are available in Supplementary material.

### 2.5 Statistical analysis

A linear mixed-effects model (LMM) was used to account for repeated measures across five time points: pre-test (T1 et T2), immediate post-treatment (T3), and two follow-up sessions at 4 weeks (T4) and 3 months post-intervention (T5). This approach accommodates within-subject dependencies and inter-individual variability by including random intercepts. LMMs are particularly suitable for single-case designs with longitudinal data, allowing for the assessment of treatment effects and their maintenance over time while maximizing statistical power despite the limited sample size. Time was included as a fixed effect, and participant was treated as a random intercept to account for inter-individual differences. Significance was determined using F-tests for the fixed effect of time and degrees of freedom were adjusted using the Satterthwaite approximation. Where a significant main effect of time was detected, post-hoc comparisons with Holm-adjusted *p*-values were performed between the baseline and each post-treatment time point to assess short- and long-term changes. The analyses were conducted in Jamovi (version 2.6.19.0), using the Mixed Models module. Given the small sample size (*n* = 5), random-effect estimates must be interpreted with caution, as their reliability is inherently reduced in very small samples. To address this limitation, descriptive data and individual trajectories are also presented to support the interpretation of treatment effects.

## 3 Results

### 3.1 Specific effects

#### 3.1.1 Trained words and pseudowords (list A)—immediate repetition

The intervention’s specific effects on immediate repetition of trained words and pseudowords are summarized in Table 4 (fixed effects) and Table 5 (random effects).

**TABLE 4:** Fixed effects estimates for immediate repetition of trained words and pseudowords.

	β	SE	95% CI	*t*(df)	*p*	BIC
**Trained words**						**180.14**
Time (intercept)	69.42	5.78	[58.10, 80.76]	12 (4)	<0.001	
T2–T1	−2	4.96	[–11.70, 7.71]	–0.4 (16)	0.692	
T3–T1	25.8	4.96	[16.10, 35.51]	5.21 (16)	<0.001	
T4–T1	24.8	4.96	[15.10, 34.51]	5 (16)	<0.001	
T5–T1	24	4.96	[14.30, 33.71]	4.84 (16)	<0.001	
**Trained pseudowords**						**172.46**
Time (intercept)	53.12	4.91	[43.49, 62.7]	10.82 (4)	<0.001	
T2–T1	2.8	4.06	[–5.16, 10.8]	0.69 (16)	0.689	
T3–T1	23.6	4.06	[15.64, 31.6]	5.81 (16)	<0.001	
T4–T1	19.6	4.06	[11.64, 27.6]	4.83 (16)	<0.001	
T5–T1	17.6	4.06	[9.64, 25.6]	4.33 (16)	<0.001	

**TABLE 5:** Random effects estimates for immediate repetition of trained words and pseudowords.

	SD	Variance	ICC
**Trained words**			
Participant (intercept)	12.45	155	0.72
Residual	7.84	61.4	
**Trained pseudowords**			
Participant (intercept)	10.60	112.3	0.73
Residual	6.42	41.3	

A significant main effect of time was found for immediate repetition of trained word accuracy, *F*(4, 16) = 16.4, *p* < 0.001 and immediate repetition of pseudowords accuracy *F*(4, 16) = 13.6, *p* < 0.001. The parameter estimates for time indicated a significant change in scores across time points for the immediate repetition of trained words and the immediate repetition of trained pseudowords. As shown in Table 5, variability among participants was also substantial, indicating marked inter-individual differences in performance.

**FIGURE 2:**
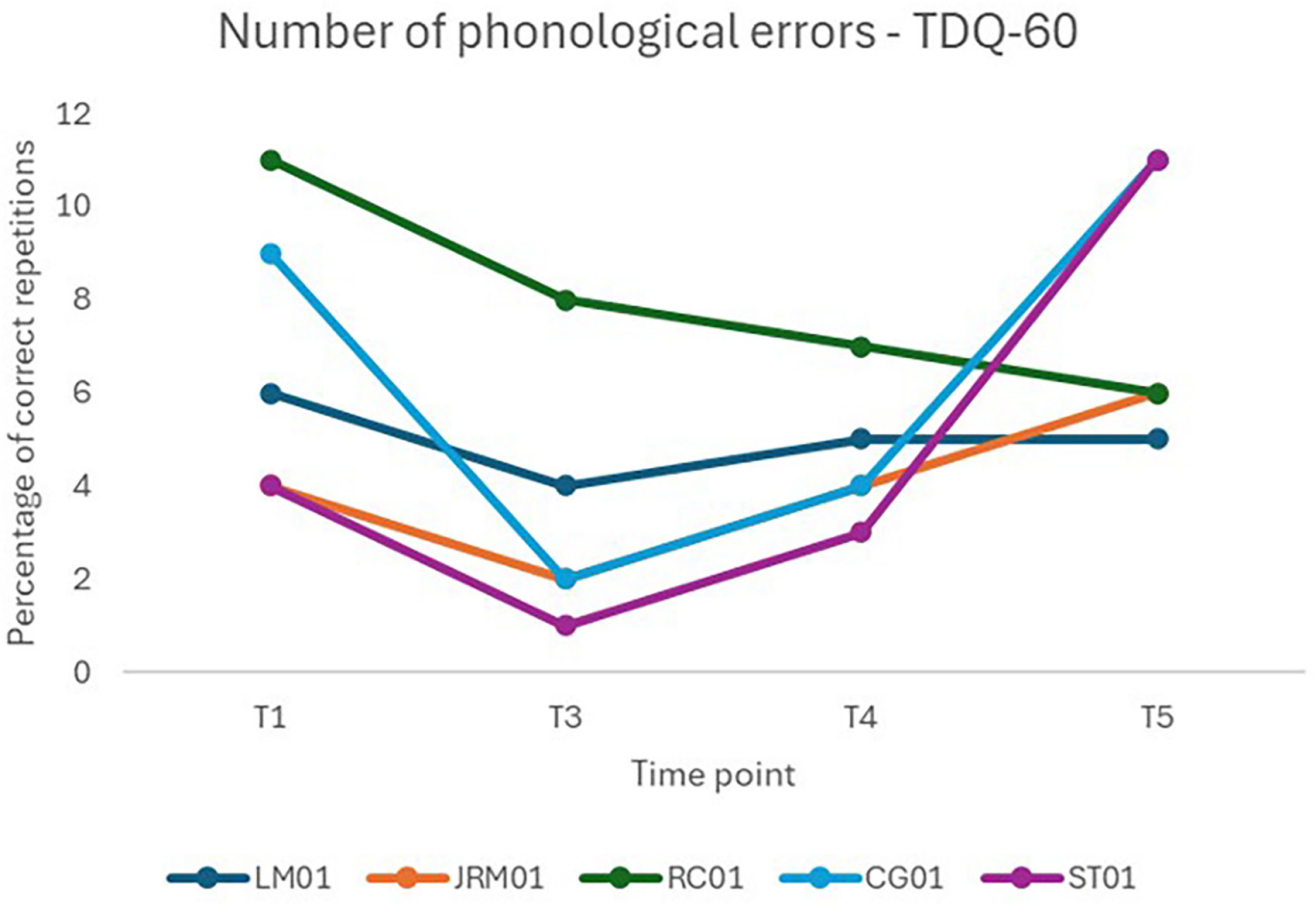
Individual performance over time on immediate repetition tasks for trained words and pseudowords.

Figure 2 illustrates a progressive increase in the accuracy of immediate repetition of trained words and pseudowords from pre-intervention (T1–T2) to post-treatment (T3), with gains maintained at follow-ups (T4 and T5). For trained words, no significant difference was observed between the two baselines [*t*(16) = 0.4, *p*_*Holm*_ = 1.000]. In contrast, performance improved significantly at T3 [*t*(16) = –5.21, *p*_*Holm*_ < 0.001], T4 [*t*(16) = –5.00, *p*_*Holm*_ < 0.001], and T5 [*t*(16) = –4.84, *p*_*Holm*_ < 0.001], with no significant decline between T3 and T4 or T5 (all *p*_*s*_ = 1.000), indicating sustained effects. A similar pattern was found for trained pseudowords. No significant difference was found between T1 and T2 [*t*(16) = –0.69, *p*_*Holm*_ = 1.000]. However, performance improved significantly at T3 [*t*(16) = –5.81, *p*_*Holm*_ < 0.001], T4 [*t*(16) = –4.83, *p*_*Holm*_ = 0.001], and T5 [*t*(16) = –4.33, *p*_*Holm*_ = 0.004] compared to T1. No significant differences were observed between T3 and follow-up time points, confirming maintenance of treatment benefits over time.

#### 3.1.2 Trained words and pseudowords (list A)—delayed repetition

The intervention’s specific effects on delayed repetition of trained words and pseudowords are reported in Table 6 (fixed effects) and Table 7 (random effects).

**TABLE 6:** Fixed effects estimates for delayed repetition of trained words and pseudowords.

	β	SE	95% CI	*t*(df)	*p*	BIC
**Trained words**						**174.72**
Time (intercept)	58.32	4.39	[49.7, 66.92]	13.29 (4)	<0.001	
T2–T1	−1.6	4.48	[−10.4, 7.19]	−0.36 (16)	0.726	
T3–T1	21.6	4.48	[12.8, 30.39]	4.82 (16)	<0.001	
T4–T1	24.4	4.48	[15.6, 33.19]	5.44 (16)	<0.001	
T5–T1	23.2	4.48	[14.4, 31.99]	5.17 (16)	<0.001	
**Trained pseudowords**						**195.54**
Time (intercept)	49.92	5.72	[38.71, 61.1]	8.73 (4)	<0.001	
T2–T1	0.2	8.04	[−15.56, 16]	0.03 (16)	0.980	
T3–T1	32.5	8.04	[16.74, 48.3]	4.04 (16)	<0.001	
T4–T1	27.7	8.04	[11.94, 43.5]	3.44 (16)	0.003	
T5–T1	19.2	8.04	[3.44, 35]	2.39 (16)	0.030	

**TABLE 7:** Random effects estimates for delayed repetition of trained words and pseudowords.

	SD	Variance	ICC
**Trained words**			
Participant (intercept)	9.29	86.2	0.63
Residual	7.09	50.3	
**Trained pseudowords**			
Participant (intercept)	11.5	131	0.45
Residual	12.7	162	

A significant effect of time was observed for delayed repetition accuracy of both trained words, *F*(4, 16) = 17.1, *p* < 0.001, and trained pseudowords, *F*(4, 16) = 7.15, *p* = 0.002. Time-related parameter estimates revealed substantial improvements across sessions for both stimulus types. Inter-individual variability was considerable for trained words, and moderate for trained pseudowords.

**FIGURE 3:**
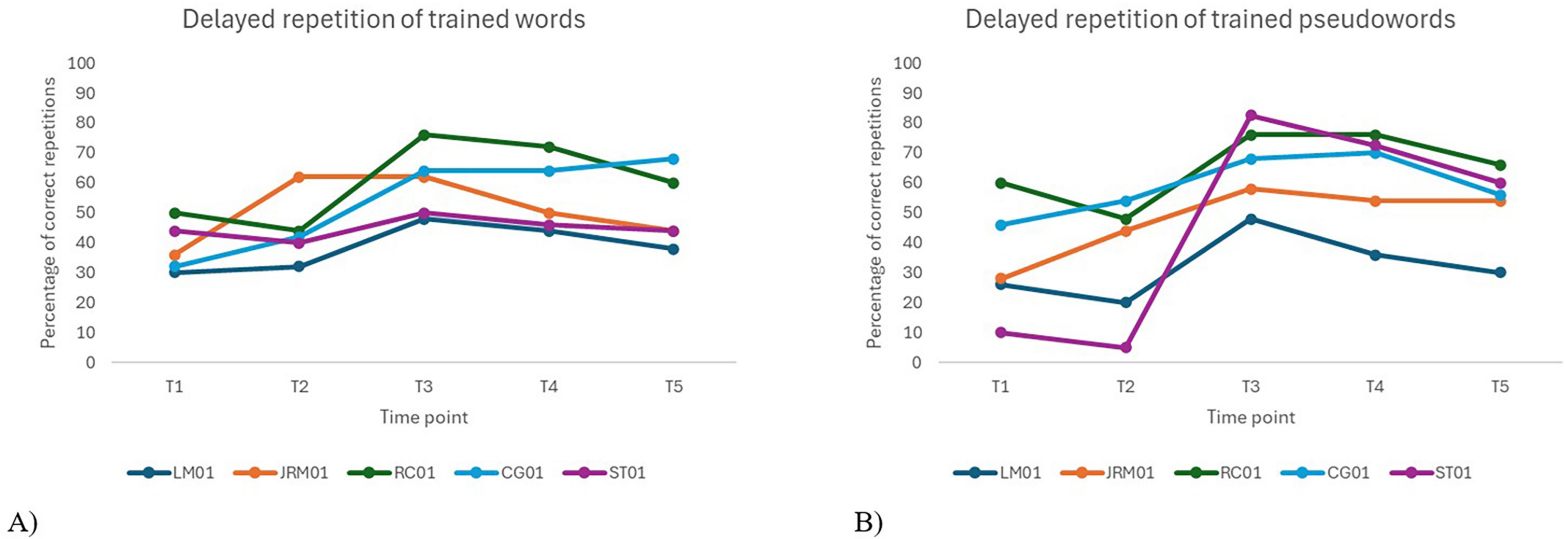
Individual performance over time on delayed repetition of trained words **(A)** and pseudowords **(B)**.

For trained words, no significant difference was observed between the two baselines [*t*(16) = 0.36, *p*_*Holm*_ = 1.000]. In contrast, significant gains were noted at T3, T4, and T5 compared to baseline (all *p*_*s*_ < 0.001), with no significant decline at follow-ups, indicating that improvements were maintained over time. For trained pseudowords, performance also remained stable at baseline [*t*(16) = –0.03, *p*_*Holm*_ = 1.000]. Significant improvements were observed at T3 (*p*_*Holm*_ = 0.009) and T4 (*p* = 0.027), but the effect at T5 did not reach statistical significance (*p*_*Holm*_ = 0.178), suggesting less robust maintenance of treatment gains over time for pseudowords compared to words. Figure 3 provides a visual representation of these patterns across participants.

### 3.2 Generalization effects

#### 3.2.1 Untrained words and pseudowords (List B)—immediate repetition

The generalized impact of the intervention on immediate repetition of untrained words and pseudowords is summarized in Table 8 (fixed effects) and Table 9 (random effects).

**TABLE 8:** Fixed effects estimates for immediate repetition of untrained words and pseudowords.

	β	SE	95% CI	*t*(df)	*p*	BIC
**Untrained trained words**						**192.08**
Time (intercept)	63.21	5.35	[52.73, 73.7]	11.82 (4)	<0.001	
T2–T1	7.6	7.34	[–6.79, 22]	1.04 (16)	.316	
T3–T1	27.3	7.34	[12.91, 41.7]	3.72 (16)	0.002	
T4–T1	23.3	7.34	[8.91, 37.7]	3.17 (16)	0.006	
T5–T1	20.81	7.34	[6.45, 35.2]	2.84 (16)	0.012	
**Untrained trained pseudowords**						**178.74**
Time (intercept)	49.68	3.78	[42.27, 57.1]	13.14 (4)	<0.001	
T2–T1	5.6	5.23	[–4.66, 15.9]	1.07 (16)	0.300	
T3–T1	21.6	5.23	[11.34, 31.9]	4.13 (16)	<0.001	
T4–T1	16.8	5.23	[6.54, 27.1]	3.21 (16)	0.005	
T5–T1	12.4	5.23	[2.14, 22.7]	2.37 (16)	0.031	

**TABLE 9:** Random effects estimates for immediate repetition of untrained words and pseudowords.

	SD	Variance	ICC
**Untrained words**			
Participant (intercept)	10.8	116	0.46
Residual	11.6	135	
**Untrained pseudowords**			
Participant (intercept)	7.6	57.8	0.46
Residual	8.27	68.5	

A significant main effect of time was observed for immediate repetition accuracy of untrained words, *F*(4, 16) = 4.92, *p* = 0.009, and untrained pseudowords, *F*(4, 16) = 5.44, *p* = 0.006.

A significant effect of time was observed for immediate repetition accuracy of both untrained words and pseudowords, with performance varying across time points. Inter-individual variability was moderate, indicating that a substantial portion of the variance was attributable to differences between participants (see Table 9).

**FIGURE 4:**
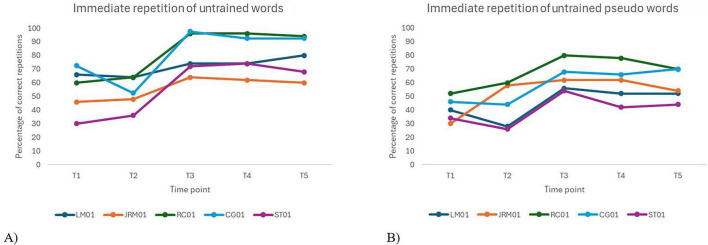
Individual performance over time on immediate repetition tasks for untrained words **(A)** and pseudowords **(B)**.

An examination of Figure 4 shows a modest improvement in immediate repetition of untrained words following immediately the intervention. As expected, no significant difference was found between the two baseline assessments (T1 and T2) for untrained words [*t*(16) = –1.04, *p*_*Holm*_ = 1.000], confirming that participants’ performance was stable before the intervention. For untrained words, performance at T3 [*t*(16) = –3.72, *p*_*Holm*_ = 0.019] and T4 [*t*(16) = –3.17, *p*_*Holm*_ = 0.053] was significantly higher than at T1, although the gain at T5 did not reach significance [*t*(16) = –2.84, *p*_*Holm*_ = 0.095]. However, no significant differences were observed between T2 and any of the post-intervention assessments (T3, T4 and T5), which raises questions about the consistency and robustness of the generalization effects beyond trained items. A similar pattern was found for untrained pseudowords. While visual inspection suggested a slight upward trend between T1 and T2, this difference was not significant [*t*(16) = –1.07, *p*_*Holm*_ = 0.901]. Significant improvements were observed from T1 to T3 [*t*(16) = –4.13, *p*_*Holm*_ = 0.008] and T4 [*t*(16) = –3.21, *p*_*Holm*_ = 0.049], but not to T5 [*t*(16) = –2.37, *p*_*Holm*_ = 0.215], suggesting a decline in effect over time. Again, no significant differences were found between T2 and the post-intervention assessments (i.e., T3, T4 and T5), further questioning the reliability of treatment generalization to untrained pseudowords.

#### 3.2.2 Untrained words and pseudowords (list B)—delayed repetition

The generalized impact of the intervention on delayed repetition of untrained words and pseudowords is summarized in Table 10 (fixed effects) and Table 11 (random effects).

**TABLE 10:** Fixed effects estimated for delayed repetition of untrained words and pseudowords.

	β	SE	95% CI	*t*(df)	*p*	BIC
**Untrained words**						**169.4**
Time (intercept)	52.72	4.13	[44.62, 60.8]	12.76 (4)	<0.001	
T2–T1	8.8	3.86	[1.24, 16.4]	2.28 (16)	0.036	
T3–T1	19.2	3.86	[11.64, 26.8]	4.98 (16)	<0.001	
T4–T1	20.4	3.86	[12.84, 28]	5.29 (16)	<0.001	
T5–T1	21.2	3.86	[13.64, 28.8]	5.50 (16)	<0.001	
**Untrained pseudowords**						**195.07**
Time (intercept)	46.88	5.91	[35.29, 58.5]	7.93 (4)	0.001	
T2–T1	1.9	7.86	[−13.51, 17.3]	0.24 (16)	0.812	
T3–T1	27.8	7.86	[12.39, 43.2]	3.54 (16)	0.003	
T4–T1	22.9	7.86	[7.49, 38.3]	2.91 (16)	0.010	
T5–T1	20.3	7.86	[4.89, 35.7]	2.58 (16)	0.020	

**TABLE 11:** Random effects estimates for delayed repetition of untrained words and pseudowords.

	SD	Variance	ICC
**Untrained words**			
Participant (intercept)	8.82	77.9	0.68
Residual	6.1	37.2	
**Untrained pseudowords**			
Participant (intercept)	12	144	0.48
Residual	12.4	154	

A statistically significant effect of time was found for delayed repetition accuracy of untrained words, *F*(4, 16) = 11.5, *p* < 0.001, as well as untrained pseudowords, *F*(4, 16) = 5.26, *p* = 0.007. The model’s fixed-effect estimates indicated robust improvements across assessment points for untrained words and pseudowords. Regarding between-subject, inter-individual variability was for untrained words and moderate for untrained pseudowords.

**FIGURE 5:**
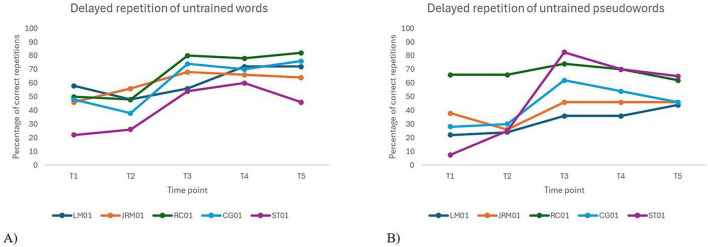
Individual performance over time on delayed repetition tasks for untrained words **(A)** and pseudowords **(B)**.

Figure 5 shows an overall improvement in delayed repetition of untrained words and pseudowords following the intervention, with effects generally maintained at follow-up. For untrained words, no significant difference was observed between pre-tests [*t*(16) = –2.28, *p*_*Holm*_ = 0.146]. However, performance significantly improved at T3 [*t*(16) = –4.98, *p*_*Holm*_ = 0.001], T4 [*t*(16) = –5.29, *p*_*Holm*_ < 0.001], and T5 [*t*(16) = –5.50, *p*_*Holm*_ < 0.001] compared to T1. Comparisons with T2 also showed significant gains at T4 [*t*(16) = –3.01, *p*_*Holm*_ = 0.050] and T5 [*t*(16) = –3.22, *p*_*Holm*_ = 0.038], confirming treatment effects. No significant decline was observed between T3 and the follow-ups, suggesting maintenance of gains over time. For untrained pseudowords, baseline performance remained stable [*t*(16) = –0.24, *p*_*Holm*_ = 1.000]. Significant improvements were observed at T3 relative to T1 [*t*(16) = –3.54, *p*_*Holm*_ = 0.027] and T2 [*t*(16) = –3.29, *p*_*Holm*_ = 0.041], but effects were not sustained at T4 or T5, as differences from T1 and T2 did not reach significance. Visual inspection of individual trajectories revealed a modest post-intervention decline in performance for one participant (ST01) in the word condition, and for three participants (CG01, RC01, ST01) in the pseudoword condition, indicating some variability in treatment maintenance at the individual level.

### 3.3 Transfer effects

#### 3.3.1 Picture naming

Transfer effects of the intervention to picture naming performance were assessed based on the number of phonological errors. Interrater agreement for phonological errors on the TDQ-60 was substantial (κ = 0.80). Results are presented in Table 12 (fixed effects) and Table 13 (random effects).

**TABLE 12:** Fixed effects estimates for the number of phonological errors in the picture-naming task.

	β	SE	95% CI	*t*(df)	*p*	BIC
						**100.81**
Time (intercept)	5.65	0.71	[4.25, 7.05]	7.91 (4)	0.001	
T3–T1	−3.4	1.56	[–6.56, −0.34]	−2.18 (16)	0.050	
T4–T1	−2.2	1.56	[−5.26, 0.86]	−1.41 (16)	0.184	
T5–T1	1	1.56	[−2.06, 4.06]	0.64 (16)	0.534	

**TABLE 13:** Random effects estimate for the number of phonological errors in the picture-naming task.

	SD	Variance	ICC
Participant (intercept)	1.01	1.02	0.14
Residual	2.47	6.1	

A marginal effect of time was observed for the number of phonological errors in the picture-naming task, *F*(3, 12) = 3.31, *p* = 0.057. Fixed-effect parameter estimates indicated a significant reduction in phonological errors immediately after the intervention (T1 vs. T3). No significant differences were observed at later points, suggesting the absence of long-term maintenance (Table 12). Inter-individual variability was relatively low, indicating that only a small proportion of the variance in phonological error rates was attributable to between-participant differences (Table 13). Given the marginal significance of the main effect, no post hoc comparisons were conducted.

**FIGURE 6:**
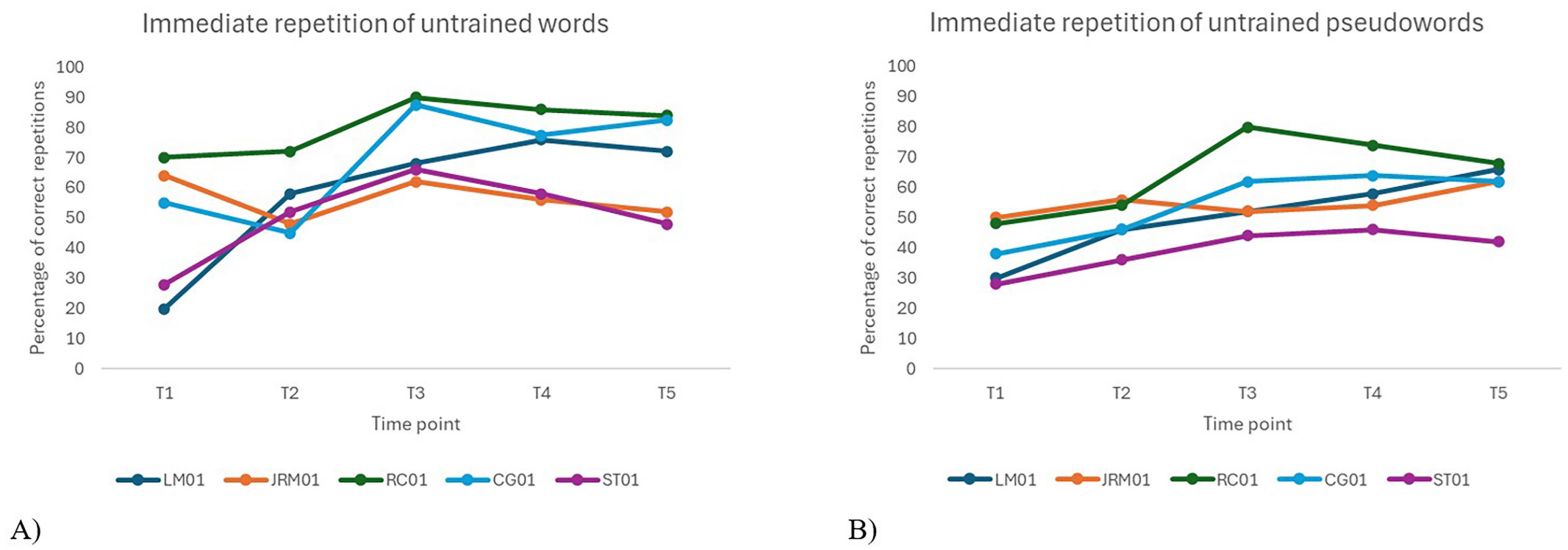
Individual performance over time on immediate repetition tasks for untrained words **(A)** and pseudowords **(B)**.

Although the effect of time on phonological errors in the picture-naming task did not reach statistical significance, Figure 6 illustrates individual trajectories over the course of the study. While a reduction in errors is apparent for all participants immediately after the intervention (T3), this trend is not consistently maintained at follow-up. Notably, error rates increased again at T5 for participants ST01 and CG01, highlighting variability in long-term treatment response.

A marginal reduction in phonological errors during picture naming was observed following the intervention, *F*(3, 12) = 3.31, *p* = 0.057, with a significant improvement noted immediately post-treatment. However, this effect was not maintained at follow-up, suggesting limited long-term transfer. Inter-individual variability was low, indicating that differences between participants accounted for little of the overall variance. As illustrated in Figure 5, most participants showed a decrease in errors at T3, but performance declined for some individuals at T5, particularly ST01 and CG01, highlighting variability in sustained benefit.

#### 3.3.2 Connected speech

Transfer effects of the intervention to connected speech (i.e., picture description of the Cookie Theft) were assessed based on the number of phonological errors. Interrater agreement for phonological errors on the Cookie Theft was κ = 0.84. Results are presented in Table 14 (fixed effects) and Table 15 (random effects).

**TABLE 14:** Fixed effects estimates for the number of phonological errors in the discursive task.

	β	SE	95% CI	*t*(df)	*p*	BIC
						**84.52**
Time (intercept)	2.95	0.53	[1.91, 3.99]	5.54 (4)	0.005	
T3–T1	−1.6	0.87	[–3.31, −0.11]	−1.83 (16)	0.092	
T4–T1	−1	0.87	[−2.71, 0.71]	−1.14 (16)	0.275	
T5–T1	<0.01	0.87	[−1.71, −1.712]	<0.01 (16)	1.000	

**TABLE 15:** Random effects estimates for the number of phonological errors in the discursive task.

	SD	Variance	ICC
Participant (intercept)	0.97	0.94	0.33
Residual	1.38	1.91	

**FIGURE 7:**
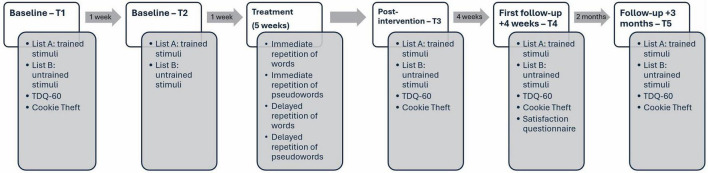
Individual performance over time on the number of phonological errors in the discursive task.

No significant main effect of time was observed, *F*(3, 12) = 1.63, *p* = 0.234, indicating no evidence of overall change in the number of phonological errors during the discursive task. Fixed-effect estimates did not reveal any statistically significant pairwise comparisons (Table 14). Inter-individual variability was moderate, with an ICC of.33, suggesting that approximately one-third of the variance in phonological errors could be attributed to differences between participants (Table 15). Individual performances across time points are illustrated in Figure 7.

#### 3.3.3 Satisfaction

Participant satisfaction with the intervention was assessed 4 weeks after treatment (T8) using an 11-item questionnaire rated on a five-point Likert scale (0 = *strongly disagree* to 4 = *strongly agree*). All five participants completed the full questionnaire. Given the ordinal nature of Likert-scale data, medians were calculated in addition to means and standard deviations. Median item scores ranged from 3 to 4, with 9 out of 11 items showing a median of 4, indicating consistently high satisfaction across participants. Only two items, clarity of activities (Q8) and perceived tolerability of the activities (Q9), yielded a median of 3, which nonetheless reflects an overall positive appraisal. Mean item scores ranged from 3.2 to 3.8 (SD = 0.45–0.89), further supporting the high acceptability and feasibility of the intervention. The highest mean scores were observed for items assessing recommendation of the treatment (Q1; *M* = 3.8, SD = 0.45) and perceived improvement in understanding of language difficulties (Q2; *M* = 3.8, SD = 0.45). Items examining the tolerability of session pace, time commitment, and treatment-related fatigue also showed elevated satisfaction (Ms = 3.6; SDs = 0.55–0.89). For illustration: Q1—“*Would you recommend this treatment to someone with similar difficulties?*”: Median = 4, *M* = 3.8 (SD = 0.45). Q3—“*Was the use of computer equipment comfortable for you?*”: Median = 4, *M* = 3.6 (SD = 0.89), with one participant reporting moderate comfort. Q8—“*Were the proposed activities clear to complete?*”: Median = 3, *M* = 3.2 (SD = 0.45), suggesting some room for improvement in task clarity. Overall, these findings demonstrate that the telerehabilitation protocol was highly acceptable, manageable, and perceived as useful by individuals with lvPPA, offering meaningful clinical value even when functional generalization remained modest. Overall, the consistently high satisfaction ratings indicate that participants perceived the protocol as clear, tolerable, and easy to integrate into their weekly routines.

## 4 Discussion

This study aimed to evaluate the effectiveness of a targeted pSTM training program delivered via teletherapy in individuals with lvPPA. Three specific objectives guided the investigation. The first was to assess whether the intervention improved repetition accuracy for trained words and pseudowords, and whether these effects were maintained at 4 weeks and 3 months post-intervention. The second objective was to determine whether these effects generalized to untrained items, both words and pseudowords, and if so, whether the generalization persisted over time. The third objective was to explore whether the intervention led to a reduction in phonological errors in more functional language tasks, such as picture naming and a discursive task, and to evaluate the maintenance of such potential improvements at follow-up. Together, these objectives aimed to provide a comprehensive assessment of the impact, generalization, and durability of pSTM training in lvPPA.

Our findings indicate significant and sustained improvements in both immediate and delayed repetition of trained items, confirming the protocol’s efficacy on its primary target. Partial generalization effects were observed, particularly in the short term, for untrained items with less consistent outcomes at the 3-month follow-up, especially for pseudowords. The results suggested that the intervention had only a limited impact on functional language performance, as evidenced by the modest changes observed in the TDQ-60 picture naming task and the Cookie Theft picture description task. These results support a specific intervention effect that is limited in scope but robust in the short- and intermediate-term.

Improvements in the repetition of trained items support the hypothesis that intensive pSTM training can yield measurable gains even in a neurodegenerative context. The greater benefit observed for words compared to pseudowords suggests a facilitative role of lexical encoding. This observation aligns with findings by Majerus et al. (2005), who emphasized the contribution of lexical representations to verbal memory maintenance. It also echoes improvements reported in post-stroke aphasia (Kalinyak-Fliszar et al., 2011; Banco et al., 2024). A novel contribution is showing that such gains are possible via teletherapy, consistent with previous feasibility studies (Theodoros, 2008; Holland et al., 2018).

The generalization effects observed for untrained items remain modest and transient. These may reflect stimulus-specific encoding, indicative of item-bound learning rather than broad skill transfer. This aligns with findings from Boo and Rose (2011) and Francis et al. (2003), who underscore the challenges of generalizing phonological gains in the absence of contextual variation or functional relevance. Repeated activation and contextualization may thus be key levers for future protocols to enhance learning transfer. Comparable trends are found in post-stroke aphasia literature, where generalization effects often require specific adjustments to be sustained (Kalinyak-Fliszar et al., 2011). These adjustments are similarly present in pSTM-oriented interventions in post-stroke aphasia that successfully demonstrated transfer, where repetition training is embedded within broader communicative or lexical contexts. Enriching training with narrative elements, functional language activities, or multimodal supports may therefore help broaden the scope of learning. This constitutes a promising avenue that was not explored in the current study.

Despite the relevance of functional language tasks for evaluating real-world communication, our study did not reveal any generalization effects on the *Cookie Theft Picture Description* task or on the TDQ-60 picture naming task. Participants produced few phonological errors at baseline, which considerably reduced the opportunity for measurable change. Moreover, the types of phonological errors observed in spontaneous or semi-structured production (e.g., phonological selection errors, anticipations, or phoneme migrations) differ from those elicited during repetition, which primarily involve failures of phonological maintenance. As a result, gains in repetition may not directly translate into improved accuracy in picture naming or discourse. Additionally, the TDQ-60 and Cookie Theft tasks may not be sensitive enough to capture subtle phonological changes, such as reduced hesitations, fewer near-miss productions, or greater stability in phoneme sequencing. These findings are consistent with previous studies targeting similar cognitive-linguistic components in non-lvPPA populations. Some studies in post-stroke aphasia found similar item-specific gains without generalization (Koenig-Bruhin and Studer-Eichenberger, 2007), whereas others reported broader gains (Kohn et al., 1990). Differences likely reflect pathology and neuroplasticity profiles. In neurodegenerative conditions such as lvPPA, generalization to functional discourse may require broader, multimodal approaches or earlier intervention stages when residual capacities are higher.

Individual trajectories revealed marked heterogeneity in treatment response. While no statistically significant change was found in TDQ-60 picture naming performance immediately post-intervention, longitudinal data showed diverging patterns. Two participants (LM01, RC01) maintained stable performance, while others showed mild decline (JRM01) or resurgence of errors (GG01, ST01) by follow-up. This interindividual variability may reflect differences in cognitive profiles (e.g., residual phonological span), fatigue levels, spontaneous compensatory strategies, or engagement. While linear mixed models helped model this dispersion, high intraclass correlation coefficients indicate that subject-level factors accounted for a substantial portion of the variance. Notably, participants CG01 and ST01, who showed the weakest maintenance at follow-up, also presented the lowest baseline cognitive-linguistic performance (MoCA scores of 10/30), contrasting with LM01, who had preserved global cognition (28/30). These differences in global cognition and processing speed likely constrained their capacity to maintain rehearsal strategies over time, potentially limiting consolidation. This interpretation aligns with Nickels et al. (2025), who reported that baseline MoCA scores predicted responsiveness to a phonological intervention in lvPPA. This pattern echoes Macoir et al. (2024), who identified contrasting repetition profiles among lvPPA patients, supporting the notion that different phonological components may be differentially impaired depending on clinical subtypes. Their findings highlight the diagnostic sensitivity of the repetition task, as well as its vulnerability to disruptions in the phonological loop and lexical representations. These factors are likely to affect treatment responsiveness.

Interestingly, in delayed repetition, untrained items, particularly words, showed significant and sustained improvement, despite no observed gain in immediate repetition. This dissociation suggests that delayed repetition may offer cognitively favorable conditions for generalization, potentially by engaging internal rehearsal mechanisms. Qualitative observations during therapy sessions support this hypothesis: JRM01 reported mentally rehearsing the sequences during the delay, while LM01 and RC01 were observed subvocalizing before producing the item post-cue. These behaviors suggest that the imposed delay may act as an active treatment component, by fostering internal phonological rehearsal. Consequently, delayed repetition may serve not merely as a consolidation test, but as a pedagogical tool that implicitly encourages patients to maintain, reactivate, or reconstruct phonological traces, thereby indirectly strengthening training effects. Future protocol versions could systematically integrate delayed repetition, or even substitute some immediate trials, to leverage this metacognitive and self-regulatory dimension.

This study is the first to investigate the restoration of phonological short-term memory in individuals with lvPPA. It offers several key clinical contributions by confirming the feasibility of delivering pSTM training via teletherapy, thereby providing a viable response to access barriers in a progressively disabling condition. Participant perceptions further reinforce this feasibility. High satisfaction ratings, particularly regarding clarity, tolerability, and overall usefulness of the remote format, suggest that the protocol was not only manageable but also accepted as meaningful and relevant by individuals with lvPPA. These perceptions support the clinical viability of teletherapy delivery for pSTM training, especially in populations facing mobility, fatigue, or access barriers. The protocol’s item complexity gradation allows dynamic adaptation to the patient’s proximal zone of development while respecting neurodegenerative constraints. This adaptable framework aligns with Koenig-Bruhin and Studer-Eichenberger (2007) and Zakariás et al. (2019), who advocate for intensive, structured, and modifiable programs that promote distributed repetition and memory consolidation. Nonetheless, treatment effects remain confined mainly to trained items, with limited generalization to broader linguistic material or spontaneous communicative contexts. Future protocols could integrate narrative and multimodal tasks to promote functional generalization.

From a methodological standpoint, several limitations must be acknowledged. First, the small sample size (five participants) and the absence of a control group limit the generalizability and evidentiary value of the findings. Second, the TDQ-60 naming task may be ill-suited to our specific research aims, as it prioritizes semantic over phonological constraints. Consequently, it cannot reliably isolate or track changes in phonological errors post-intervention. A more targeted phonological measure, such as one involving minimal pairs or phonotactically controlled stimuli, could have better captured post-treatment changes. This underlines the importance of aligning outcome measures with therapeutic targets. Additionally, no ecological communication measure was included, preventing conclusions about real-world verbal participation or autonomy. Finally, Hawthorne or task habituation effects cannot be entirely ruled out, especially in a longitudinal design without test rotation. From a broader outcomes perspective, our battery only partially overlaps with domains recommended in the COS-PPA core outcome set (Volkmer and Hardy, 2025); future studies should incorporate additional measures (e.g., participation, caregiver-reported outcomes) to enhance comparability and strengthen clinical relevance.

Immediate next steps include integrating this protocol with other validated therapeutic approaches, such as lexico-semantic interventions like Semantic Feature Analysis (Boyle and Coelho, 1995), to address both anomia and phonological errors in a combined, targeted manner. This hybridization could more effectively link phonological improvements to residual semantic resources, enhancing communication outcomes. Next, a larger-scale group study is needed to confirm the robustness of these preliminary findings, ideally followed by a randomized controlled trial. Incorporating narrative or interactive components (e.g., guided storytelling, scripted dialogues, or conversational exercises) could support generalization in ecological contexts. In addition, expanding the protocol to other linguistic and cultural populations would enhance clinical adaptability and cross-cultural validity. On the methodological front, adding neurofunctional measures (fMRI) would allow for a deeper exploration of neuroplastic mechanisms underpinning behavioral changes. Moreover, emerging evidence suggests that a patient’s baseline functioning level may be a key predictor of treatment efficacy, particularly in neurodegenerative conditions. Meyer et al. (2016), in a written-language intervention in PPA, reported strong correlations between baseline performance on the *trained task itself* (writing) and treatment gains, underscoring the importance of preserved task-specific abilities prior to intervention. This is distinct from global measures such as the MoCA, but together these findings highlight that both task-specific capacity and broader cognitive functioning contribute to treatment responsiveness in neurodegenerative conditions. This suggests a potential prophylactic effect, whereby early intervention, while certain capacities are still preserved, may help stabilize or prolong function. These findings reinforce the need for personalized, early-stage protocols tailored to the patient’s cognitive profile. Finally, a promising line of inquiry involves examining phonological self-regulation in lvPPA. While some patients retain partial error-detection abilities, spontaneous self-correction is often lacking. Understanding how these individuals initiate, adjust, or sustain phonological recall strategies could enhance therapeutic autonomy. Such approaches have shown promise in post-traumatic aphasia (Jeffay et al., 2023) and could support the development of metacognitive phonological strategies tailored to neurodegenerative profiles.

## 5 Conclusion

This study demonstrates the feasibility and efficacy of intensive, remotely delivered phonological short-term memory training in individuals with lvPPA. Treatment effects were robust and sustained for trained items, while generalization remained partial, especially in delayed repetition. In the present study, no substantial generalization effects were observed in functional language tasks, including picture description and picture naming tasks, suggesting that the intervention’s impact may remain task specific. High interindividual variability and the influence of intrinsic factors highlight the value of personalized, early-stage interventions. These findings support the development of future protocols combining targeted phonological training with more functional tasks and call for further investigation into the role of self-regulation in therapy.

## Data Availability

The raw data supporting the conclusions of this article will be made available by the authors, without undue reservation.

## References

[B1] Baddeley A. (1992). Working memory. *Science* 255 556–559. 10.1126/Science.1736359 1736359

[B2] Baddeley A. Gathercole S. Papagno C. (1998). The phonological loop as a language learning device. *Psychol. Rev.* 105 158–173. 10.1037/0033-295X.105.1.158 9450375

[B3] Banco E. Diana L. Casati C. Tesio L. Vallar G. Bolognini N. (2024). Rehabilitation of post-stroke aphasia by a single protocol targeting phonological, lexical, and semantic deficits with speech output tasks: A randomized controlled trial. *Eur. J. Phys. Rehabil. Med.* 61:9. 10.23736/S1973-9087.24.08576-9 39704642 PMC11920753

[B4] Barbeau E. Didic M. Tramoni E. Felician O. Joubert S. Sontheimer A. (2004). Evaluation of visual recognition memory in MCI patients. *Neurology* 62 1317–1322. 10.1212/01.WNL.0000120548.24298.DB 15111668

[B5] Bereau M. Magnin E. Nicolier M. Berthet L. Dariel E. Ferreira S. (2016). Left prefrontal repetitive transcranial magnetic stimulation in a logopenic variant of primary progressive aphasia: A case report. *Eur. Neurol.* 76 12–18. 10.1159/000447399 27344155

[B6] Berthier M. L. Dávila G. Green-Heredia C. Torres I. M. Juárez y Ruiz de Mier R. (2014). Massed sentence repetition training can augment and speed up recovery of speech production deficits in patients with chronic conduction aphasia receiving donepezil treatment. *Aphasiology* 28 188–218. 10.1080/02687038.2013.861057

[B7] Bolly C. T. Crible L. Degand L. Uygur-Distexhe D. (2017). “Towards a model for discourse marker annotation in spoken french: From potential to feature-based discourse markers,” in *Discourse markers, pragmatic markers and modal particles: New perspectives*, eds Fedriani C. Sanso A. (Amsterdam: Benjamins), 71–98.

[B8] Boo M. Rose M. L. (2011). The efficacy of repetition, semantic, and gesture treatments for verb retrieval and use in Broca’s aphasia. *Aphasiology* 25 154–175. 10.1080/02687031003743789

[B9] Boyle M. Coelho C. A. (1995). Application of semantic feature analysis as a treatment for aphasic dysnomia. *Am. J. Speech-Lang. Pathol.* 4 94–98. 10.1044/1058-0360.0404.94

[B10] Cadório I. Lousada M. Martins P. Figueiredo D. (2017). Generalization and maintenance of treatment gains in primary progressive aphasia (PPA): A systematic review. *Intern. J. Lang. Commun. Disord.* 52 543–560. 10.1111/1460-6984.12310 28120406

[B11] Conca F. Esposito V. Giusto G. Cappa S. F. Catricalà E. (2022). Characterization of the logopenic variant of primary progressive aphasia: A systematic review and meta-analysis. *Ageing Res. Rev.* 82:101760. 10.1016/j.arr.2022.101760 36244629

[B12] Darrigrand B. Mazaux J. M. (2000). L’échelle de communication verbale de Bordeaux: Une évaluation des compétences communicatives des personnes aphasiques. *Glossa* 4–14.

[B13] Davies K. Howe T. Small J. Hsiung G. Y. R. (2025). “I used to be a storyteller”: The perspectives of people with primary progressive aphasia on the communication needs for themselves and their family members. *Aphasiology* 39 710–731. 10.1080/02687038.2024.2373430

[B14] Dial H. R. Hinshelwood H. A. Grasso S. M. Hubbard H. I. Gorno-Tempini M. L. Henry M. L. (2019). Investigating the utility of teletherapy in individuals with primary progressive aphasia. *Clin. Intervent. Aging* 14 453–471. 10.2147/CIA.S178878 30880927 PMC6394239

[B15] Francis D. Clark N. Humphreys G. (2003). The treatment of an auditory working memory deficit and the implications for sentence comprehension abilities in mild “receptive” aphasia. *Aphasiology* 17 723–750. 10.1080/02687030344000201

[B16] Goodglass H. Kaplan E. (1972). *Boston diagnostic aphasia examination*, 2nd Edn. Philadelphia, PA: Lea and Febiger.

[B17] Gorno-Tempini M. L. Hillis A. E. Weintraub S. Kertesz A. Mendez M. Cappa S. F. (2011). Classification of primary progressive aphasia and its variants. *Neurology* 76 1006–1014. 10.1212/WNL.0b013e31821103e6 21325651 PMC3059138

[B18] Henry M. L. Wilson S. M. Babiak M. C. Mandelli M. L. Beeson P. M. Miller Z. A. (2016). Phonological processing in primary progressive aphasia. *J. Cogn. Neurosci.* 28 210–222. 10.1162/jocn_a_00901 26544920 PMC4855288

[B19] Holland R. Johns S. L. Woollams A. M. (2018). The impact of phonological versus semantic repetition training on generalisation in chronic stroke aphasia reflects differences in dorsal pathway connectivity. *Neuropsychol. Rehabil.* 28 548–567. 10.1080/09602011.2016.1190384 27291388

[B20] Jeffay E. Ponsford J. Harnett A. Janzen S. Patsakos E. Douglas J. (2023). INCOG 2.0 guidelines for cognitive rehabilitation following traumatic brain injury, part III: Executive functions. *J. Head Trauma Rehabil.* 38 52–64. 10.1097/HTR.0000000000000834 36594859

[B21] Kalinyak-Fliszar M. Kohen F. Martin N. (2011). Remediation of language processing in aphasia: Improving activation and maintenance of linguistic representations in (verbal) short-term memory. *Aphasiology* 25 1095–1131. 10.1080/02687038.2011.577284 22791930 PMC3393127

[B22] Kaplan E. F. Goodglass H. Weintraub S. (1983). *The boston naming test*, 2nd Edn. Philadelphia, PA: Lea & Febiger.

[B23] Koenig-Bruhin M. Studer-Eichenberger F. (2007). Therapy of short-term memory disorders in fluent aphasia: A single case study. *Aphasiology* 21 448–458. 10.1080/02687030600670593

[B24] Kohn S. E. Smith K. L. Arsenault J. K. (1990). The remediation of conduction aphasia via sentence repetition: A case study. *Br. J. Disord. Commun.* 25 45–60. 10.3109/13682829009011962 1695853

[B25] Lavoie M. Bier N. Laforce R. Jr. Macoir J. (2020). Improvement in functional vocabulary and generalization to conversation following a self-administered treatment using a smart tablet in primary progressive aphasia. *Neuropsychol. Rehabil.* 10.1080/09602011.2019.1570943 30714482

[B26] Macoir J. Beaudoin C. Bluteau J. Potvin O. Wilson M. A. (2018). TDQ-60–a color picture-naming test for adults and elderly people: Validation and normalization data. *Aging Neuropsychol. Cogn.* 25 753–766. 10.1080/13825585.2017.1372355 28853339

[B27] Macoir J. Gauthier C. Jean C. Potvin O. (2016). BECLA, a new assessment battery for acquired deficits of language: Normative data from Quebec-French healthy younger and older adults. *J. Neurol. Sci.* 361 220–228. 10.1016/j.jns.2016.01.004 26810547

[B28] Macoir J. Sauvageau V. M. Boissy P. Tousignant M. Tousignant M. (2017). In-home synchronous telespeech therapy to improve functional communication in chronic poststroke aphasia: Results from a quasi-experimental study. *Telemed. e-Health* 23 630–639. 10.1089/tmj.2016.0235 28112589

[B29] Macoir J. Laforce R. Lavoie M. (2024). The impact of phonological short-term memory impairment on verbal repetition in the logopenic variant of primary progressive aphasia. *Aging Neuropsychol. Cogn.* 31, 723–741. 10.1080/13825585.2023.2249198 37615549

[B30] Majerus S. (2013). Language repetition and short-term memory: An integrative framework. *Front. Hum. Neurosci.* 7:357. 10.3389/fnhum.2013.00357 23874280 PMC3709421

[B31] Majerus S. Van der Kaa M. A. Renard C. Van der Linden M. Poncelet M. (2005). Treating verbal short-term memory deficits by increasing the duration of temporary phonological representations: A case study. *Brain Lang.* 95 174–175. 10.1016/j.bandl.2005.07.094

[B32] Mandelli M. L. Lorca-Puls D. L. Lukic S. Montembeault M. Gajardo-Vidal A. Licata A. (2023). Network anatomy in logopenic variant of primary progressive aphasia. *Hum. Brain Mapp.* 44 4390–4406. 10.1002/hbm.26388 37306089 PMC10318204

[B33] Martin N. Saffran E. M. (1997). Language and auditory-verbal short-term memory impairments: Evidence for common underlying processes. *Cogn. Neuropsychol.* 14 641–682. 10.1080/026432997381402

[B34] Martin N. Saffran E. M. (2002). The relationship of input and output phonological processing: An evaluation of models and evidence to support them. *Aphasiology* 16 107–150. 10.1080/02687040143000447

[B35] Martin N. Saffran E. M. Dell G. S. (1996). Recovery in deep dysphasia: Evidence for a relation between auditory–verbal STM capacity and lexical errors in repetition. *Brain Lang.* 52 83–113. 10.1006/brln.1996.0005 8741977

[B36] Mesulam M. M. Rogalski E. J. Wieneke C. Hurley R. S. Geula C. Bigio E. H. (2014). Primary progressive aphasia and the evolving neurology of the language network. *Nat. Rev. Neurol.* 10 554–569. 10.1038/nrneurol.2014.159 25179257 PMC4201050

[B37] Meyer A. M. Getz H. R. Brennan D. M. Hu T. M. Friedman R. B. (2016). Telerehabilitation of anomia in primary progressive aphasia. *Aphasiology* 30 483–507. 10.1080/02687038.2015.1081142 27087732 PMC4831866

[B38] Meyer A. M. Snider S. F. Campbell R. E. Friedman R. B. (2015). Phonological short-term memory in logopenic variant primary progressive aphasia and mild Alzheimer’s disease. *Cortex* 71 183–189. 10.1016/j.cortex.2015.07.003 26232551 PMC4521400

[B39] Nasreddine Z. S. Phillips N. A. Bédirian V. Charbonneau S. Whitehead V. Collin I. (2005). The Montreal Cognitive Assessment, MoCA: A brief screening tool for mild cognitive impairment. *J. Am. Geriatrics Soc.* 53 695–699. 10.1111/j.1532-5415.2005.53221.x 15817019

[B40] Neary D. Snowden J. S. Gustafson L. Passant U. Stuss D. Black S. A. S. A. (1998). Frontotemporal lobar degeneration: A consensus on clinical diagnostic criteria. *Neurology* 51 1546–1554. 10.1212/WNL.51.6.1546 9855500

[B41] Newhart M. Davis C. Kannan V. Heidler-Gary J. Cloutman L. Hillis A. E. (2009). Therapy for naming deficits in two variants of primary progressive aphasia. *Aphasiology* 23 823–834. 10.1080/02687030802661762

[B42] Nickels K. Beeson P. M. Kielar A. (2025). Addressing phonological deficit in primary progressive aphasia with behavioral intervention and transcranial direct current stimulation. *J. Speech Lang. Hear. Res.* 68 2348–2385. 10.1044/2024_JSLHR-24-00250 40227131

[B43] Nickels L. Howard D. (1995). Phonological errors in aphasic naming: Comprehension. *Monitor. Lexical. Cortex* 31 209–237. 10.1016/S0010-9452(13)80360-7 7555004

[B44] Pagnoni I. Gobbi E. Premi E. Borroni B. Binetti G. Cotelli M. (2021). Language training for oral and written naming impairment in primary progressive aphasia: A review. *Trans. Neurodegenerat.* 10:24. 10.1186/s40035-021-00248-z 34266501 PMC8282407

[B45] Sohlberg M. M. McLaughlin K. A. Pavese A. Heidrich A. Posner M. I. (2000). Evaluation of attention process training and brain injury education in persons with acquired brain injury. *J. Clin. Exp. Neuropsychol.* 22 656–676. 10.1076/1380-3395(200010)22:5;1-9;FT656 11094401

[B46] Taylor B. Bocchetta M. Shand C. Todd E. G. Chokesuwattanaskul A. Crutch S. J. (2025). Data-driven neuroanatomical subtypes of primary progressive aphasia. *Brain* 148 955–968. 10.1093/brain/awae314 39374849 PMC11884653

[B47] Theodoros D. G. (2008). Telerehabilitation for service delivery in speech-language pathology. *J. Telemed. Telecare* 14 221–224. 10.1258/jtt.2007.007044 18632993

[B48] Tippett D. C. Hillis A. E. Tsapkini K. (2015). Treatment of primary progressive aphasia. *Curr. Treatment Opt. Neurol.* 17:34. 10.1007/s11940-015-0362-5 26062526 PMC4600091

[B49] Trebbastoni A. Raccah R. De Lena C. Zangen A. Inghilleri M. (2013). Repetitive deep transcranial magnetic stimulation improves verbal fluency and written language in a patient with primary progressive aphasia-logopenic variant (LPPA). *Brain Stimul.* 6 545–553. 10.1016/j.brs.2012.09.014 23122915

[B50] Tsapkini K. Hillis A. E. (2013). Spelling intervention in post-stroke aphasia and primary progressive aphasia. *Behav. Neurol.* 26 55–66. 10.3233/BEN-2012-110240 22713403 PMC3459145

[B51] Volkmer A. Hardy C. J. (2025). An international core outcome set for primary progressive aphasia (COS-PPA): Consensus-based. *J. Alzheimer’s Dement.* 21:e14362. 10.1002/alz.14362 39535361 PMC11781257

[B52] Wechsler D. (1997). *WAIS-3. WMS-3: Wechsler adult intelligence scale, Wechsler memory scale: Technical manual.*

[B53] Wechsler D. (2008). *Wechsler adult intelligence scale–fourth edition (WAIS-IV) [database record].* APA PsycTests. 10.1037/t15169-000

[B54] Whitwell J. L. Jones D. T. Duffy J. R. Strand E. A. Machulda M. M. Przybelski S. A. (2015). Working memory and language network dysfunctions in logopenic aphasia: A task-free fMRI comparison with Alzheimer’s dementia. *Neurobiol. Aging* 36 1245–1252. 10.1016/j.neurobiolaging.2014.12.013 25592958 PMC4346438

[B55] Zakariás L. Kelly H. Salis C. Code C. (2019). The methodological quality of short-term/working memory treatments in poststroke aphasia: A systematic review. *J. Speech Lang. Hear. Res.* 62 1979–2001. 10.1044/2018_JSLHR-L-18-0057 31120801

