# Supplementary material for: Effectiveness of a teletherapy-based phonological short-term memory training in reducing phonological impairments in the logopenic variant of primary progressive aphasia: a multiple case study
Source: Front Hum Neurosci. 2025 Dec 18;19:1724345. doi: 10.3389/fnhum.2025.1724345 (PMC12756969; doi:10.3389/fnhum.2025.1724345)

Supplementary Material

Summary

1. List of trained and untrained words 1

2. List of trained and untrained pseudowords 27

3. English version of the detailed intervention procedure: phonological short-term memory training 46

4. French version of the detailed intervention procedure: phonological short-term memory training 51

1. List of trained and untrained words

List of two-syllable trained words (A)

Immediate repetition

Word	cgram	freqfilms2	nbsyll	cv-cv	S1	S2
forêt	NOM	1,16	2	CV-CV	CV	CV
gouttières	NOM	0,52	2	CV-CYVC	CV	CYVC
flambeau	NOM	2,66	2	CCV-CV	CCV	CV
aimés	ADJ	0,5	2	V-CV	V	CV
coincés	ADJ	1,19	2	CYV-CV	CYV	CV
payant	ADJ	1,23	2	CV-YV	CV	YV
biquet	NOM	0,54	2	CV-CV	CV	CV
casquettes	NOM	1,09	2	CVC-CVC	CVC	CVC
latin	ADJ	1,78	2	CV-CV	CV	CV
roman	ADJ	1,09	2	CV-CV	CV	CV
	**MEAN**	**1,19**				
	**SD**	**0,70**				

<table>
<tr><th colspan="2">DISTRIBUTION SYLL STRUCT</th><th>N</th><th>%</th></tr>
<tr><td>V</td><td>1</td><td rowspan="4">15</td><td rowspan="4">80</td></tr>
<tr><td>CV</td><td>13</td></tr>
<tr><td>VC</td><td>0</td></tr>
<tr><td>YV</td><td>1</td></tr>
<tr><td>CCV</td><td>1</td><td rowspan="5">4</td><td rowspan="5">20</td></tr>
<tr><td>CVC</td><td>2</td></tr>
<tr><td>CCYV</td><td>0</td></tr>
<tr><td>CYV</td><td>0</td></tr>
<tr><td>CYVC</td><td>1</td></tr>
</table>

Delayed repetition

Word	cgram	freqfilms2	nbsyll	cv-cv	S1	S2
adroit	ADJ	1,13	2	V-CCYV	V	CCYV
renom	NOM	1,15	2	CV-CV	CV	CV
soucoupes	NOM	1,23	2	CV-CVC	CV	CVC
tonnerres	NOM	0,5	2	CV-CVC	CV	CVC
artères	NOM	2	2	VC-CVC	VC	CVC
têtus	ADJ	0,55	2	CV-CV	CV	CV
sujets	ADJ	0,55	2	CV-CV	CV	CV
bossus	NOM	0,55	2	CV-CV	CV	CV
courants	NOM	2,89	2	CV-CV	CV	CV
foulée	NOM	0,5	2	CV-CV	CV	CV
	**MEAN**	**1,11**				

**SD**	**0,79**

**DISTRIBUTION SYLL STRUCT**		N	**%**
V	1	16	**80**
CV	14		
VC	1		
YV	0		
CCV	0	4	**20**
CVC	3		
CCYV	1		
CYV	0		
CYVC	0		

List of two-syllable untrained words (B)

Immediate repetition

Word	cgram	freqfilms2	nbsyll	cv-cv	S1	S2
nuisible	ADJ	0,56	2	CYV-CVCC	CYV	CVCC
futées	ADJ	0,55	2	CV-CV	CV	CV
fâcheux	ADJ	2,13	2	CV-CV	CV	CV
secrète	NOM	0,54	2	CV-CCVC	CV	CCVC
convives	NOM	0,53	2	CV-CVC	CV	CVC
géante	ADJ	2,81	2	CV-VC	CV	VC
cuvée	NOM	0,55	2	CV-CV	CV	CV
verrue	NOM	0,66	2	CV-CV	CV	CV
défis	NOM	2,02	2	CV-CV	CV	CV
goujon	NOM	0,5	2	CV-CV	CV	CV
	**MEAN**	**1,09**				
	**SD**	**0,88**				

**DISTRIBUTION SYLL STRUCT**		N	**%**
V	0	16	**80**
CV	15		
VC	1		
YV	0		
CCV	0	4	**20**
CVC	1		
CCYV	0		
CCVC	1		
CYVC	0		
CVCC	1		

CYV	1		

Delayed repetition

Word	cgram	freqfilms2	nbsyll	cv-cv	S1	S2
boudin	NOM	2,49	2	CV-CV	CV	CV
marées	NOM	1,1	2	CV-CV	CV	CV
sereins	ADJ	0,5	2	CV-CV	CV	CV
copeaux	NOM	0,57	2	CV-CV	CV	CV
brutaux	ADJ	0,92	2	CCV-CV	CCV	CV
lointain	NOM	0,81	2	CYV-CV	CYV	CV
connues	ADJ	0,91	2	CV-CV	CV	CV
compresse	NOM	0,76	2	CV-CCVC	CV	CCVC
dauphins	NOM	2,63	2	CV-CV	CV	CV
fauteuils	NOM	2,1	2	CV-CVY	CV	CVY
	**MEAN**	**1,28**				
	**SD**	**0,81**				

DISTRIBUTION SYLL STRUCT		N	%
V	0	16	**80**
CV	16		
VC	0		
YV	0		
CCV	1	4	**20**
CVC	0		
CVY	1		
CYV	1		
CYVC	0		
CCVC	1		

List of three-syllable trained words (A)

Immediate repetition

Word	cgram	freqfilms2	nbsyll	cv-cv	S1	S2	S3
culinaire	ADJ	1,14	3	CV-CV-CVC	CV	CV	CVC
budgétaires	ADJ	0,53	3	CVC-CV-CVC	CVC	CV	CVC
lavabo	NOM	2,56	3	CV-CV-CV	CV	CV	CV
estomacs	NOM	0,5	3	VC-CV-CV	VC	CV	CV
écuyer	NOM	1,18	3	V-CYV-YV	V	CYV	YV
urticaire	NOM	1,22	3	VC-CV-CVC	VC	CV	CVC
agités	ADJ	0,51	3	V-CV-CV	V	CV	CV
enchantés	ADJ	0,55	3	V-CV-CV	V	CV	CV
orchidées	NOM	1,9	3	VC-CV-CV	VC	CV	CV

abricot	NOM	0,5	3	V-CCV-CV	V	CCV	CV
	**MEAN**	**1,06**					
	**SD**	**0,70**					

**DISTRIBUTION SYLL STRUCT**		N	%
V	4	24	**80**
CV	16		
VC	3		
YV	1		
CCV	1	6	**20**
CVC	4		
CCYV	0		
CYV	1		
CYVC	0		

Delayed repetition

Word	cgram	freqfilms2	nbsyll	cv-cv	S1	S2	S3
nourrisson	NOM	1,13	3	CV-CV-CV	CV	CV	CV
envoyés	NOM	1,16	3	V-CYV-YV	V	CYV	YV
établi	ADJ	1,22	3	V-CV-CCV	V	CV	CCV
distinguée	ADJ	1,23	3	CVC-CV-CV	CVC	CV	CVC
déguisée	ADJ	0,5	3	CV-CV-CV	CV	CV	CV
maternelle	ADJ	1,91	3	CV-CVC-CVC	CV	CVC	CVC
satanée	ADJ	2,08	3	CV-CV-CV	CV	CV	CV
arrogant	ADJ	2,98	3	V-CV-CV	V	CV	CV
envolée	NOM	0,55	3	V-CV-CV	V	CV	CV
épineux	ADJ	0,5	3	V-CV-CV	V	CV	CV
	**MEAN**	**1,33**					
	**SD**	**0,80**					

**DISTRIBUTION SYLL STRUCT**		N	%
V	5	23	**79**
CV	17		
VC	0		
YV	1		
CCV	1	6	**21**
CVC	4		
CCYV	0		
CYV	1		
CYVC	0		

List of three-syllable untrained words (B)

Immediate repetition

Word	cgram	freqfilms2	nbsyll	cv-cv	S1	S2	S3
cachetons	NOM	0,56	3	CV-CV-CV	CV	CV	CV
citadin	NOM	0,56	3	CV-CV-CV	CV	CV	CV
honorables	ADJ	2,13	3	V-CV-CVCC	V	CV	CVCC
ratatouille	NOM	0,54	3	CV-CV-CVY	CV	CV	CVY
ultrasons	NOM	0,53	3	VC-CCV-CV	VC	CCV	CV
gouvernail	NOM	2,81	3	CV-CVC-CVY	CV	CVC	CVY
vainement	ADV	0,55	3	CV-CV-CV	CV	CV	CV
embryon	NOM	0,6	3	V-CCV-YV	V	CCV	YV
parmesan	NOM	0,66	3	CVC-CV-CV	CVC	CV	CV
honoré	ADJ	2,02	3	V-CV-CV	V	CV	CV
	**MEAN**	**1,10**					
	**SD**	**0,87**					

DISTRIBUTION SYLL STRUCT		N	%
V	3	23	**77**
CV	18		
VC	1		
YV	1		
CCV	2	7	**23**
CVC	2		
CCYV	0		
CYV	0		
CVY	2		
CVCC	1		

Delayed repetition

Word	cgram	freqfilms2	nbsyll	cv-cv	S1	S2	S3
attractions	NOM	2,48	3	V-CCVC-CYV	V	CCVC	CYV
démenti	NOM	1,01	3	CV-CV-CV	CV	CV	CV
entouré	ADJ	0,57	3	V-CV-CV	V	CV	CV
bâillement	NOM	0,5	3	CV-YV-CV	CV	YV	CV
négligent	ADJ	0,92	3	CV-CCV-CV	CV	CCV	CV
effarant	ADJ	0,81	3	V-CV-CV	V	CV	CV
découverte	ADJ	0,91	3	CV-CV-CVCC	CV	CV	CVCC

répandue	ADJ	0,76	3	CV-CV-CV	CV	CV	CV
littéraire	ADJ	2,64	3	CV-CV-CVC	CV	CV	CVC
capitaines	NOM	2,11	3	CV-CV-CVC	CV	CV	CVC
	**MEAN**	**1,27**					
	**SD**	**0,81**					

**DISTRIBUTION SYLL STRUCT**		N	%
V	3	24	**80**
CV	20		
VC	0		
YV	1		
CCV	1	6	**20**
CVCC	1		
CVC	2		
CYV	1		
CYVC	0		
CCVC	1		

List of four-syllable trained words (A)

Immediate repetition

Word	nbsyll	cgram	freqfilms2	cv-cv	S1	S2	S3	S4
divinement	4	ADV	1,14	CV-CV-CV-CV	CV	CV	CV	CV
publicités	4	NOM	1,18	CV-CCV-CV-CV	CV	CCV	CV	CV
républicaine	4	ADJ	1,22	CV-CV-CCV-CVC	CV	CV	CCV	CVC
philosophies	4	NOM	0,5	CV-CV-CV-CV	CV	CV	CV	CV
pénalités	4	NOM	0,51	CV-CV-CV-CV	CV	CV	CV	CV
conformité	4	NOM	0,52	CV-CVC-CV-CV	CV	CVC	CV	CV
implantation	4	NOM	0,53	V-CCV-CV-CYV	V	CCV	CV	CYV
inattendue	4	ADJ	1,9	V-CV-CV-CV	V	CV	CV	CV
interrupteur	4	NOM	2,58	V-CV-CVC-CVC	V	CV	CVC	CVC
malhonnêteté	4	NOM	0,5	CV-CV-CVC-CV	CV	CV	CVC	CV
		**MEAN**	**1,06**					
		**SD**	**0,71**					

**DISTRIBUTION SYLL STRUCT**		N	%
V	3	24	**80**
CV	21		
VC	0		
YV	0		
CCV	3	6	**20**
CVC	3		

CCYV	0		
CYV	0		
CYVC	0		

Delayed repetition

Word	nbsyll	cgram	freqfilms2	cv-cv	S1	S2	S3	S4
bouleversée	4	ADJ	1,13	CV-CV-CVC-CV	CV	CV	CVC	CV
déraisonnable	4	ADJ	1,15	CV-CV-CV-CVCC	CV	CV	CV	CVCC
carrosserie	4	NOM	1,22	CV-CV-CV-CV	CV	CV	CV	CV
chirurgical	4	ADJ	1,23	CV-CVC-CV-CVC	CV	CVC	CV	CVC
viennoiserie	4	NOM	0,52	CYV-CYV-CV-CV	CYV	CYV	CV	CV
animateur	4	NOM	1,9	V-CV-CV-CVC	V	CV	CV	CVC
brièvement	4	ADV	1,92	CCV-YV-CV-CV	CCV	YV	CV	CV
brutalement	4	ADV	2,89	CCV-CV-CV-CV	CCV	CV	CV	CV
prématuré	4	NOM	0,55	CCV-CV-CV-CV	CCV	CV	CV	CV
endommagée	4	ADJ	0,57	V-CV-CV-CV	V	CV	CV	CV
		**MEAN**	**1,31**					
		**SD**	**0,75**					

DISTRIBUTION SYLL STRUCT		N	%
V	2	23	**77**
CV	20		
VC	0		
YV	1		
CCV	3	7	**23**
CVC	2		
CCYV	0		
CYV	2		
CYVC	0		

List of four-syllable untrained words (B)

Immediate repetition

Word	nbsyll	cgram	freqfilms2	cv-cv	S1	S2	S3	S4
privilégiée	4	ADJ	0,56	CCV-CV-CV-CYV	CCV	CV	CV	CYV
invocation	4	NOM	0,55	V-CV-CV-CYV	V	CV	CV	CYV
autorisées	4	ADJ	0,55	V-CV-CV-CV	V	CV	CV	CV
verbalement	4	ADV	0,53	CVC-CV-CV-CV	CVC	CV	CV	CV
félicité	4	NOM	2,1	CV-CV-CV-CV	CV	CV	CV	CV

antiquités	4	NOM	2,8	V-CV-CV-CV	V	CV	CV	CV
industrielles	4	ADJ	0,55	V-CVC-CCV-YVC	V	CVC	CCV	YVC
illimitée	4	ADJ	0,6	V-CV-CV-CV	V	CV	CV	CV
mythologique	4	ADJ	0,66	CV-CV-CV-CVC	CV	CV	CV	CVC
habileté	4	NOM	2,03	V-CV-CV-CV	V	CV	CV	CV
		**MEAN**	**1,09**					
		**SD**	**0,86**					

**DISTRIBUTION SYLL STRUCT**		N	**%**
V	6	32	**80**
CV	26		
VC	0		
YV	0		
CCV	2	8	**20**
CVC	3		
CCYV	0		
CYV	2		
CYVC	0		
YVC	1		

Delayed repetition

**Word**	**nbsyll**	**cgram**	**freqfilms2**	**cv-cv**	**S1**	**S2**	**S3**	**S4**
sévèrement	4	ADV	2,46	CV-CV-CV-CV	CV	CV	CV	CV
réactionnaires	4	ADJ	1,01	CV-VC-CYV-CVC	CV	VC	CYV	CVC
hôtellerie	4	NOM	0,57	V-CV-CV-CV	V	CV	CV	CV
incontrôlables	4	ADJ	0,57	V-CV-CCV-CVCC	V	CV	CCV	CVCC
enveloppé	4	ADJ	0,86	V-CV-CV-CV	V	CV	CV	CV
hypothermie	4	NOM	0,8	V-CV-CVC-CV	V	CV	CVC	CV
astronomie	4	NOM	0,91	VC-CCV-CV-CV	V	CCV	CV	CV
fatalité	4	NOM	2,66	CV-CV-CV-CV	CV	CV	CV	CV
échappement	4	NOM	1,93	V-CV-CV-CV	V	CV	CV	CV
incontournable	4	ADJ	0,5	V-CV-CVC-CVCC	V	CV	CVC	CVCC
		**MEAN**	**1,23**					
		**SD**	**0,81**					

**DISTRIBUTION SYLL STRUCT**		N	**%**
V	7	32	**80**
CV	24		
VC	1		
YV	0		
CCV	2	8	**20**

CVC	3		
CCYV	0		
CYV	1		
CYVC	0		
YVC	0		
CVCC	2		

List of five-syllable trained words (A)

Immediate repetition

Word	nbsyll	cgram	freqfilms2	cv-cv	S1	S2	S3	S4	S5
excessivement	5	ADV	1,14	VC-CV-CV-CV-CV	VC	CV	CV	CV	CV
international	5	NOM	0,53	V-CVC-CV-CYV-CVC	V	CVC	CV	CYV	CVC
décomposition	5	NOM	2,58	CV-CV-CV-CV-CYV	CV	CV	CV	CV	CYV
relativité	5	NOM	0,5	CV-CV-CV-CV-CV	CV	CV	CV	CV	CV
spécifiquement	5	ADV	1,18	CCV-CV-CV-CV-CV	CCV	CV	CV	CV	CV
camaraderie	5	NOM	1,22	CV-CV-CV-CV-CV	CV	CV	CV	CV	CV
canalisation	5	NOM	0,51	CV-CV-CV-CV-CYV	CV	CV	CV	CV	CYV
archéologique	5	ADJ	0,52	VC-CV-V-CV-CVC	VC	CV	V	CV	CVC
supériorité	5	NOM	2	CV-CV-CYV-CV-CV	CV	CV	CYV	CV	CV
assaisonnement	5	NOM	0,5	V-CV-CV-CV-CV	V	CV	CV	CV	CV
		**MEAN**	**1,07**						
		**SD**	**0,73**						

**DISTRIBUTION SYLL STRUCT**		N	**%**
V	3	42	**84**
CV	37		
VC	2		
YV	0		
CCV	1	8	**16**
CVC	3		
CYV	4		

Delayed repetition

Word	nbsyll	cgram	freqfilms2	cv-cv	S1	S2	S3	S4	S5
officieusement	5	ADV	1,13	V-CV-CYV-CV-CV	V	CV	CYV	CV	CV
amicalement	5	ADV	1,15	V-CV-CV-CV-CV	V	CV	CV	CV	CV
multinationale	5	NOM	1,22	CVC-CV-CV-CYV-CVC	CVC	CV	CV	CYV	CVC
précipitation	5	NOM	1,9	CCV-CV-CV-CV-CYV	CCV	CV	CV	CV	CYV
indéfiniment	5	ADV	2,08	V-CV-CV-CV-CV	V	CV	CV	CV	CV
ravitaillement	5	NOM	2,66	CV-CV-CV-YV-CV	CV	CV	CV	YV	CYV
recommandation	5	NOM	2,89	CV-CV-CV-CV-CYV	CV	CV	CV	CV	CYV
coordinateur	5	NOM	0,54	CV-VC-CV-CV-CVC	CV	VC	CV	CV	CVC
illégalité	5	NOM	0,55	V-CV-CV-CV-CV	V	CV	CV	CV	CV
régularité	5	NOM	0,55	CV-CV-CV-CV-CV	CV	CV	CV	CV	CV
		**MEAN**	**1,47**						
		**SD**	**0,87**						

**DISTRIBUTION SYLL STRUCT**		N	**%**
V	4	41	**82**
CV	35		
VC	1		
YV	1		
CCV	1	9	**18**
CVC	3		
CYV	5		

List of five-syllable untrained words (B)

Immediate repetition

Word	nbsyll	cgram	freqfilms2	cv-cv	S1	S2	S3	S4	S5
commémorative	5	ADJ	0,56	CV-CV-CV-CV-CVC	CV	CV	CV	CV	CVC
indépendamment	5	ADV	0,55	V-CV-CV-CV-CV	V	CV	CV	CV	CV
dangereusement	5	ADV	2,13	CV-CV-CV-CV-CV	CV	CV	CV	CV	CV

articulation	5	NOM	0,54	VC-CV-CV-CV-CYV	VC	CV	CV	CV	CYV
interplanétaire	5	ADJ	0,53	V-CVC-CCV-CV-CVC	V	CVC	CCV	CV	CVC
caractéristiques	5	NOM	2,81	CV-CVC-CV-CVC-CVC	CV	CVC	CV	CVC	CVC
aboutissement	5	NOM	0,5	V-CV-CV-CV-CV	V	CV	CV	CV	CV
réalisatrice	5	NOM	0,6	CV-V-CV-CV-CCVC	CV	V	CV	CV	CCVC
luminosité	5	NOM	0,66	CV-CV-CV-CV-CV	CV	CV	CV	CV	CV
organisations	5	NOM	2,02	VC-CV-CV-CV-CYV	VC	CV	CV	CV	CYV
		**MEAN**	**1,09**						
		**SD**	**0,87**						

**DISTRIBUTION SYLL STRUCT**		N	**%**
V	4	40	**80**
CV	34		
VC	2		
YV	0		
CCV	1	10	**20**
CVC	6		
CYV	2		
CCVC	1		

Delayed repetition

**Word**	**nbsyll**	**cgram**	**freqfilms2**	**cv-cv**	**S1**	**S2**	**S3**	**S4**	**S5**
inimaginable	5	ADJ	2,48	V-CV-CV-CV-CVCC	V	CV	CV	CV	CVCC
pacificateur	5	NOM	1,1	CV-CV-CV-CV-CVC	CV	CV	CV	CV	CV
classification	5	NOM	0,57	CCV-CV-CV-CV-CYV	CCV	CV	CV	CV	CYV
imaginations	5	NOM	0,5	V-CV-CV-CV-CYV	V	CV	CV	CV	CYV
avertissements	5	NOM	0,92	V-CVC-CV-CV-CV	V	CVC	CV	CV	CV
manipulateur	5	NOM	0,81	CV-CV-CV-CV-CVC	CV	CV	CV	CV	CVC
rectification	5	NOM	0,91	CVC-CV-CV-CV-CYV	CVC	CV	CV	CV	CYV
légitimité	5	NOM	0,76	CV-CV-CV-CV-CV	CV	CV	CV	CV	CV
unanimité	5	NOM	2,63	V-CV-CV-CV-CV	V	CV	CV	CV	CV

interrogatoires	5	NOM	2,1	V-CV-CV-CV-CYVC	V	CV	CV	CV	CYVC
		**MEAN**	**1,28**						
		**SD**	**0,81**						

**DISTRIBUTION SYLL STRUCT**		N	**%**
V	5	41	**82**
CV	36		
VC	0		
YV	0		
CCV	1	9	**18**
CVC	3		
CYV	3		
CVCC	1		
CYVC	1		

List of six-syllable trained words (A)

Immediate repetition

Word 1	Word 2	cgram	freqWord1	freqWord2	nbsyll	cv-cv	S1	S2	S3	S4	S5	S6
épopée	carburant	NOM+ADJ	1,13	1,14	6	V-CV-CV-CVC-CV-CV	V	CV	CV	CVC	CV	CV
frigidaire	libéré	NOM+ADJ	1,16	1,16	6	CCV-CV-CVC-CV-CV-CV	CCV	CV	CVC	CV	CV	CV
cohérent	galaxies	ADJ+NOM	1,23	1,23	6	CV-V-CV-CV-CVC-CV	CV	V	CV	CV	CVC	CV
évolué	acérées	ADJ+ADJ	0,5	0,51	6	V-CV-CYV-V-CV-CV	V	CV	CYV	V	CV	CV
dramatique	balancé	NOM+ADJ	0,5	0,51	6	CCV-CV-CVC-CV-CV-CV	CCV	CV	CVC	CV	CV	CV
sucrier	mosaïque	NOM+NOM	0,51	0,53	6	CV-CCV-YV-CV-CV-VC	CV	CCV	YV	CV	CV	VC
insensée	déguisé	ADJ+ADJ	1,92	1,93	6	V-CV-CV-CV-CV-CV	V	CV	CV	CV	CV	CV
vigoureux	enfantine	ADJ+ADJ	2,05	2,07	6	CV-CV-CV-V-CV-CVC	CV	CV	CV	V	CV	CVC
endormi	livraisons	ADJ+NOM	2,95	2,98	6	V-CVC-CV-CV-CCV-CV	V	CVC	CV	CV	CCV	CV
parental	ambiguë	ADJ+ADJ	0,5	0,5	6	CV-CV-CVC-V-CV-CV	CV	CV	CVC	V	CV	CV
		**MEAN**	**1,25**									
		**SD**	**0,81**									

**DISTRIBUTION SYLL STRUCT**		N	**%**
V	8	48	**80**
CV	38		
VC	1		
YV	1		
CCV	4	12	**20**
CVC	7		
CCYV	0		
CYV	1		
CYVC	0		

Delayed repetition

Word 1	Word 2	cgram	freqWord1	freqWord2	nbsyll	cv-cv	S1	S2	S3	S4	S5	S6
continents	dégradant	NOM+ADJ	1,13	1,13	6	CV-CV-CV-CV-CCV-CV	CV	CV	CV	CV	CCV	CV
distractions	côtelette	NOM+NOM	1,18	1,18	6	CVC-CCVC-CYV-CV-CV-CVC	CVC	CCVC	CYV	CV	CV	CVC
émotive	radicaux	ADJ+ADJ	1,22	1,22	6	V-CV-CVC-CV-CV-CV	V	CV	CVC	CV	CV	CV
finitions	pétunia	NOM+NOM	0,5	0,5	6	CV-CV-CYV-CV-CV-CYV	CV	CV	CYV	CV	CV	CYV
abonnés	arbalète	NOM+NOM	0,52	0,52	6	V-CV-CV-VC-CV-CVC	V	CV	CV	VC	CV	CVC
arrachée	boutonneux	ADJ+ADJ	0,55	0,55	6	V-CV-CV-CV-CV-CV	V	CV	CV	CV	CV	CV
graffiti	moucheron	NOM+NOM	1,93	1,93	6	CCV-CV-CV-CV-CV-CV	CCV	CV	CV	CV	CV	CV
excessif	demeuré	ADJ+NOM	2,05	2,06	6	VC-CV-CVC-CV-CV-CV	VC	CV	CVC	CV	CV	CV
entité	dinosaures	NOM+NOM	2,12	2,14	6	V-CV-CV-CV-CV-CVC	V	CV	CV	CV	CV	CVC
chérubin	renommée	NOM+NOM	1,89	1,89	6	CV-CV-CV-CV-CV-CV	CV	CV	CV	CV	CV	CV
		**MEAN**	**1,31**									
		**SD**	**0,64**									

**DISTRIBUTION SYLL STRUCT**		N	**%**
V	4	48	**81**
CV	42		
VC	2		
YV	0		
CCV	2	11	**19**
CVC	6		
CCYV	0		
CYV	3		
CYVC	0		

List of six-syllable untrained words (B)

Immediate repetition

Word 1	Word 2	cgram	freqWord1	freqWord2	nbsyll	cv-cv	S1	S2	S3	S4	S5	S6
invaincu	licencié	ADJ+ADJ	0,5	0,5	6	V-CV-CV-CV-CV-CYV	V	CV	CV	CV	CV	CYV
maniement	mécontente	NOM+ADJ	0,5	0,5	6	CV-CV-CV-CV-CV-CVC	CV	CV	CV	CV	CV	CVC
radiation	épicier	NOM+NOM	2,71	2,73	6	CV-CYV-CYV-V-CV-CYV	CV	CYV	CYV	V	CV	CYV
désigné	argentées	ADJ+ADJ	0,51	0,52	6	CV-CV-CV-VC-CV-CV	CV	CV	CV	VC	CV	CV
excentrique	dépendance	ADJ+NOM	1,97	2,03	6	VC-CV-CCVC-CV-CV-CVC	VC	CV	CCVC	CV	CV	CVC
imprévue	épuisette	ADJ+NOM	1,56	0,51	6	V-CCV-CV-V-CYV-CVC	V	CCV	CV	V	CYV	CVC
millénaire	offenseur	NOM+NOM	2,1	0,55	6	CV-CV-CVC-V-CV-CVC	CV	CV	CVC	V	CV	CVC
détriment	souterrains	NOM+NOM	0,57	1,6	6	CV-CCV-CV-CV-CV-CV	CV	CCV	CV	CV	CV	CV
arrêté	maréchal	ADJ+NOM	2,73	2,67	6	V-CV-CV-CV-CV-CVC	V	CV	CV	CV	CV	CVC
parental	marathon	ADJ+ADJ	0,5	1,98	6	CV-CV-CVC-CV-CV-CV	CV	CV	CV	CV	CV	CV
		**MEAN**	**1,36**									
		**SD**	**0,93**									

**DISTRIBUTION SYLL STRUCT**		N	**%**
V	6	46	**77**
CV	38		
VC	2		
YV	0		
CCV	2	14	**23**
CVC	6		
CCYV	0		
CYV	5		
CVY	0		
CCVC	1		

Delayed repetition

Word 1	Word 2	cgram	freqWord1	freqWord2	nbsyll	cv-cv	S1	S2	S3	S4	S5	S6
traumatique	répandu	ADJ+ADJ	0,56	0,51	6	CCV-CV-CVC-CV-CV-CV	CCV	CV	CVC	CV	CV	CV
conteneurs	baromètre	NOM+NOM	0,55	0,56	6	CV-CV-CVC-CV-CV-CVCC	CV	CV	CVC	CV	CV	CVCC
arrosé	infirmier	ADJ+ADJ	0,56	1,37	6	V-CV-CV-V-CVC-CYV	V	CV	CV	V	CVC	CYV
demeurés	simagrées	NOM+NOM	0,55	0,9	6	CV-CV-CV-CV-CV-CCV	CV	CV	CV	CV	CV	CCV
pointilleux	enfantin	ADJ+ADJ	0,89	0,92	6	CYV-CV-YV-V-CV-CV	CYV	CV	YV	V	CV	CV
assuré	concubine	ADJ+NOM	1,56	1,56	6	V-CV-CV-CV-CV-CVC	V	CV	CV	CV	CV	CVC
jardinage	randonnée	NOM+NOM	1,65	1,74	6	CVC-CV-CVC-CV-CV-CV	CVC	V	CVC	CV	CV	CV
électeurs	dureté	NOM+NOM	2,96	1,56	6	V-CVC-CVC-CV-CV-CV	V	CVC	CVC	CV	CV	CV
lunatique	millimètre	ADJ+NOM	1,58	1,58	6	CV-CV-CVC-CV-CV-CVCC	CV	CV	CVC	CV	CV	CVCC
broderie	digitale	NOM+ADJ	0,93	0,93	6	CCV-CV-CV-CV-CV-CVC	CCV	CV	CV	CV	CV	CVC
		**MEAN**	**1,17**									
		**SD**	**0,62**									

**DISTRIBUTION SYLL STRUCT**		N	**%**
V	6	42	**76**
CV	35		
VC	0		
YV	1		
CCV	3	13	**24**
CVCC	2		
CVC	6		
CYV	2		
CYVC	0		
CCVC	0		

List of seven-syllable trained words (A)

Immediate repetition

Word 1	Word 2	Word 3	cgram	freqWord 1	freqWord 2	freqWord 3	nbsyll	cv-cv	S1	S2	S3	S4	S5	S6	S7
bisons	laquais	ralliement	NOM+NOM+NOM	1,16	1,15	1,16	7	CV-CV-CV-CV-CV-CV-CV	CV	CV	CV	CV	CV	CV	CV
secousses	jarret	indiqué	NOM+NOM+ADJ	1,15	1,16	1,1	7	CV-CVC-CV-CV-V-CV-CV	CV	CVC	CV	CV	V	CV	CV
codé	lentille	minerai	ADJ+NOM+NOM	1,22	1,22	1,21	7	CV-CV-CV-CVY-CV-CV-CV	CV	CV	CV	CVY	CV	CV	CV
bouleaux	dresseur	balancé	NOM+NOM+ADJ	0,5	0,5	0,51	7	CV-CV-CCV-CVC-CV-CV-CV	CV	CV	CCV	CVC	CV	CV	CV
errance	hautain	rossignols	NOM+ADJ+NOM	0,51	0,51	0,5	7	V-CVC-V-CV-CV-CV-CVC	V	CVC	V	CV	CV	CV	CVC
caries	clochers	fonderie	NOM+NOM+NOM	0,51	0,51	0,51	7	CV-CV-CCV-CV-CV-CV-CV	CV	CV	CCV	CV	CV	CV	CV
taillé	usées	variation	ADJ+ADJ+NOM	0,5	0,5	0,5	7	CV-YV-V-CV-CV-CYV-CYV	CV	YV	V	CV	CV	CYV	CYV
senteur	turbines	répandu	NOM+NOM+ADJ	0,51	0,51	0,51	7	CV-CVC-CVC-CVC-CV-CV-CV	CV	CVC	CVC	CVC	CV	CV	CV
phobie	aisé	cascadeur	NOM+ADJ+NOM	1,43	1,44	1,88	7	CV-CV-V-CV-CVC-CV-CVC	CV	CV	V	CV	CVC	CV	CVC
volants	marraine	vigilante	ADJ+NOM+ADJ	2,47	2,52	1,04	7	CV-CV-CV-CVC-CV-CV-CVC	CV	CV	CV	CVC	CV	CV	CVC
			**MEAN**	**0,96**											
			**SD**	**0,57**											

<table>
<tr><th colspan="2">DISTRIBUTION SYLL STRUCT</th><th>N</th><th>%</th></tr>
<tr><td>V</td><td>5</td><td rowspan="4">54</td><td rowspan="4">78</td></tr>
<tr><td>CV</td><td>48</td></tr>
<tr><td>VC</td><td>0</td></tr>
<tr><td>YV</td><td>1</td></tr>
<tr><td>CCV</td><td>2</td><td>15</td><td>22</td></tr>
</table>

CVC	11		
CCYV	0		
CYV	2		
CYVC	0		
CCVC	0		
YVC	0		
CVCC	0		

Delayed repetition

Word 1	Word 2	Word 3	cgram	freqWord 1	freqWord 2	freqWord 3	nbsyll	cv-cv	S1	S2	S3	S4	S5	S6	S7
coupole	divins	pharaons	NOM+ADJ+NOM	1,13	1,12	1,12	7	CV-CVC-CV-CV-CV-CV-V	CV	CVC	CV	CV	CV	CV	V
marrants	pointus	insensés	ADJ+ADJ+ADJ	1,33	0,95	1,09	7	CV-CV-CYV-CV-V-CV-CV	CV	CV	CYV	CV	V	CV	CV
tulipes	mollets	dégoûtants	NOM+NOM+ADJ	1,1	1,11	1,33	7	CV-CVC-CV-CV-CV-CV-CV	CV	CVC	CV	CV	CV	CV	CV
denrée	pénombre	paillasson	NOM+NOM+NOM	1,23	1,22	0,93	7	CV-CV-CVCC-CV-YV-CV	CV	CV	CV	CVCC	CV	YV	CV
ferrée	sommets	calories	ADJ+NOM+NOM	2,27	2,29	1,28	7	CV-CV-CV-CV-CV-CV-CV	CV	CV	CV	CV	CV	CV	CV
battue	fortunes	ingrédient	NOM+NOM+NOM	1,25	1,27	1,48	7	CV-CV-CVC-CVC-V-CCV-CYV	CV	CV	CVC	CVC	V	CCV	CYV
homards	laitier	antilope	NOM+NOM+NOM	1,18	1,18	0,73	7	V-CVC-CV-CYV-V-CV-CVC	V	CVC	CV	CYV	V	CV	CVC
noté	pendus	pesanteur	ADJ+NOM+ADJ	0,97	0,97	1,12	7	CV-CV-CV-CV-CV-CV-CVC	CV	CV	CV	CV	CV	CV	CVC
trachée	cyniques	dérangée	ADJ+ADJ+ADJ	0,97	0,8	0,88	7	CCV-CV-CV-CVC-CV-CV-CV	CCV	CV	CV	CVC	CV	CV	CV
touchée	perron	délégués	ADJ+NOM+NOM	0,97	1,27	1,56	7	CV-CV-CV-CV-CV-CV-CV-CV	CV	CV	CV	CV	CV	CV	CV
			**MEAN**	**1,20**											
			**SD**	**0,35**											

DISTRIBUTION SYLL STRUCT		N	%
V	5	56	**84**
CV	50		
VC	0		
YV	1		
CCV	2	11	**16**
CVC	8		
CCYV	0		
CYV	0		
CYVC	0		
CCVC	0		
CVCC	1		

List of seven-syllable untrained words (B)

Immediate repetition

Word 1	Word 2	Word 3	cgram	freqWord 1	freqWord 2	freqWord 3	nbsyll	cv-cv	S1	S2	S3	S4	S5	S6	S7
licornes	alpins	consentant	NOM+ADJ+ADJ	0,57	0,58	0,51	7	CV-CVCC-VC-CV-CV-CV-CV	CV	CVCC	VC	CV	CV	CV	CV
burin	tilleuls	épanouie	NOM+NOM+ADJ	0,57	0,56	0,56	7	CV-CV-CV-YVC-V-CV-CYV	CV	CV	CV	YVC	V	CV	CYV
trousseau	salons	invité	NOM+NOM+ADJ	2,13	2,19	2,23	7	CCV-CV-CV-CV-V-CV-CV	CCV	CV	CV	CV	V	CV	CV
signés	versant	assorti	ADJ+NOM+ADJ	0,54	0,53	0,53	7	CVC-CV-CV-CV-V-CVC-CV	CVC	CV	CV	CV	V	CVC	CV
virtuose	épis	garnements	NOM+NOM+NOM	0,52	0,52	0,52	7	CVC-CYVC-V-CV-CVC-CV-CV	CVC	CYVC	V	CV	CVC	CV	CV
ménages	pipeau	convaincant	NOM+NOM+ADJ	1,91	1,84	2,86	7	CV-CVC-CV-CV-CV-CV-CV	CV	CVC	CV	CV	CV	CV	CV
conquis	tondu	journalier	ADJ+ADJ+ADJ	0,52	0,51	0,52	7	CV-CV-CV-CV-CVC-CV-CYV	CV	CV	CV	CV	CVC	CV	CYV
gaîté	képis	inchangée	NOM+NOM+ADJ	0,55	0,55	0,54	7	CV-CV-CV-CV-V-CV-CV	CV	CV	CV	CV	V	CV	CV

barrés	essaim	gigantesque	ADJ+NOM+ADJ	0,69	0,69	0,72	7	CV-CV-V-CV-CV-CV-CVCC	CV	CV	V	CV	CV	CV	CVCC
cubains	déchet	fondateurs	NOM+NOM+NOM	1,27	1,27	2,64	7	CV-CV-CV-CV-CV-CV-CVC	CV	CV	CV	CV	CV	CV	CVC
			**MEAN**	**1,00**											
			**SD**	**0,75**											

DISTRIBUTION SYLL STRUCT		N
V	5	56
CV	49	
VC	2	
YV	0	
CCV	1	14
CVC	7	
CCYV	0	
CYV	2	
CYVC	1	
CCVC	0	
YVC	1	
CVCC	2	

Delayed repetition

Word 1	Word 2	Word 3	cgram	freqWord 1	freqWord 2	freqWord 3	nbsyl l	cv-cv	S1	S2	S3	S4	S5	S6	S7
patin	repli	rangement	NOM+NOM+NOM	1,12	1,12	1,14	7	CV-CV-CV-CCV-CV-CV-CV	CV	CV	CV	CCV	CV	CV	CV
naguère	uni	congelé	ADV+ADJ+ADJ	1,16	1,17	1,16	7	CV-CVC-V-CV-CV-CV-CV	CV	CVC	V	CV	CV	CV	CV
manies	bouché	patinoire	NOM+ADJ+NOM	1,21	1,22	1,21	7	CV-CVC-V-CV-CV-CV-CYVC	CV	CV	CV	CV	CV	CV	CYVC
fermées	dindon	frénésie	NOM+ADJ+NOM	1,23	1,23	1,23	7	CV-CV-CVC-CV-CCV-CV-CV	CV	CV	CVC	CV	CCV	CV	CV

termites	absents	canadien	NOM+ADJ+NOM	1,88	1,91	1,86	7	CVC-CVC-VC-CV-CV-CV-CYV	CVC	CVC	VC	CV	CV	CV	CYV
logé	bilans	fracturé	ADJ+NOM+ADJ	0,5	0,5	0,5	7	CV-CV-CV-CV-CCVC-CV-CV	CV	CV	CV	CV	CCVC	CV	CV
furie	rouleaux	bûcheron	NOM+NOM+NOM	2,05	2,06	2,13	7	CV-CV-CV-CV-CV-CV-CV	CV	CV	CV	CV	CV	CV	CV
juriste	pâtés	volontaire	NOM+NOM+NOM	1,95	1,96	2,98	7	CV-CVCC-CV-CV-CV-CV-CVC	CV	CVCC	CV	CV	CV	CV	CVC
pipette	guindé	pétillant	NOM+ADJ+ADJ	0,54	0,55	0,55	7	CV-CVC-CV-CV-CV-CV-YV	CV	CVC	CV	CV	CV	CV	YV
grillées	montant	médiévale	ADJ+ADJ+ADJ	0,5	0,5	0,5	7	CCV-YV-CV-CV-CV-CYV-CVC	CCV	YV	CV	CV	CV	CYV	CVC

**MEAN**	**1,25**
**SD**	**0,65**

**DISTRIBUTION SYLL STRUCT**		N
V	1	55
CV	51	
VC	1	
YV	2	
CCV	3	14
CVC	7	
CCYV	0	
CYV	1	
CYVC	1	
CCVC	1	
CVCC	1	

List of eight-syllable trained words (A)

Immediate repetition

Word 1	Word 2	Word 3	cgram	freqWord 1	freqWord 2	freqWord 3	nbsyl 1	cv-cv	S1	S2	S3	S4	S5	S6	S7	S8

cécité	fraternels	unie	ADJ+ADJ+ADJ	1,12	1,13	1,17	8	CV-CV-CV-CCV-CVC-CVC-V-CV	CV	CV	CV	CCV	CVC	CVC	V	CV
coccinelle	différend	actuelles	NOM+NOM+ADJ	1,15	1,15	1,14	8	CVC-CV-CVC-CV-CV-CV-VC-CYVC	CVC	CV	CVC	CV	CV	CV	VC	CYVC
raffinée	horizons	rivale	ADJ+NOM+NOM	1,23	1,24	1,23	8	CV-CV-CV-CV-CV-CV-CV-CVC	CV	CV	CV	V	CV	CV	CV	CVC
réjouissances	apparat	roseaux	NOM+NOM+NOM	0,5	0,51	0,5	8	CV-CYV-CVC-V-CV-CV-CV-CV	CV	CYV	CVC	V	CV	CV	CV	CV
crampons	rareté	gonflée	NOM+NOM+ADJ	0,51	0,51	0,5	8	CV-CV-CV-CV-CV-CV-CV-CCV	CCV	CV	CV	CV	CV	CV	CV	CCV
chambellan	communal	limon	NOM+ADJ+NOM	0,53	0,53	0,53	8	CV-CV-CV-CV-CV-CVC-CV-CV	CV	CV	CV	CV	CV	CVC	CV	CV
endurci	avenant	soumis	ADJ+ADJ+ADJ	0,5	0,52	0,5	8	V-CVC-CV-V-CV-CV-CV-CV	V	CVC	CV	V	CV	CV	CV	CV
attirant	effrayée	rangée	ADJ+ADJ+NOM	2,59	2,51	2,54	8	V-CCV-YV-CV-CV-CV-CV-CV	V	CCV	YV	CV	CV	CV	CV	CV
alchimiste	animés	bandeau	NOM+ADJ+NOM	2,65	2,66	2,34	8	VC-CV-CVCC-V-CV-CV-CV-CV	VC	CV	CVCC	V	CV	CV	CV	CV
saccadés	conquérants	basé	ADJ+NOM+ADJ	0,51	0,52	0,51	8	CV-CV-CV-CV-CV-CV-CV-CV	CV	CV	CV	CV	CV	CV	CV	CV
			**MEAN**	**1,12**												
			**SD**	**0,79**												

<table>
<tr><th colspan="2">DISTRIBUTION SYLL STRUCT</th><th>N</th><th>%</th></tr>
<tr><td>V</td><td>7</td><td rowspan="4">56</td><td rowspan="4">79</td></tr>
<tr><td>CV</td><td>46</td></tr>
<tr><td>VC</td><td>2</td></tr>
<tr><td>YV</td><td>1</td></tr>
<tr><td>CCV</td><td>4</td><td rowspan="7">15</td><td rowspan="7">21</td></tr>
<tr><td>CVC</td><td>8</td></tr>
<tr><td>CCYV</td><td>0</td></tr>
<tr><td>CYV</td><td>1</td></tr>
<tr><td>CYVC</td><td>1</td></tr>
<tr><td>CCVC</td><td>0</td></tr>
<tr><td>YVC</td><td>0</td></tr>
</table>

CVCC	1		

Delayed repetition

Word 1	Word 2	Word 3	cgram	freqWord 1	freqWord 2	freqWord 3	nbsyl 1	cv-cv	S1	S2	S3	S4	S5	S6	S7	S8
accordé	cabanon	véreux	NOM+NOM+ADJ	1,22	1,26	1,23	8	V-CVC-CV-CV-CV-CV-CV-CV	V	CVC	CV	CV	CV	CV	CV	CV
décidée	défenseurs	enjeux	ADJ+NOM+NOM	1,15	1,15	1,16	8	CV-CV-CV-CV-CV-CVC-V-CV	CV	CV	CV	CV	CV	CVC	V	CV
convaincue	grondement	porteuse	ADJ+NOM+ADJ	1,18	1,18	0,98	8	CV-CV-CV-CCV-CV-CV-CV-CV	CV	CV	CV	CCV	CV	CV	CVC	CVC
mutilé	pellicules	pichet	ADJ+NOM+NOM	1,22	1,23	1,21	8	CV-CV-CV-CV-CV-CVC-CV-CV	CV	CV	CV	CV	CV	CVC	CV	CV
agité	quotidien	rigide	ADJ+NOM+ADJ	2,9	2,95	2,96	8	V-CV-CV-CV-CYV-CV-CVC	V	CV	CV	CV	CV	CYV	CV	CVC
dérobée	funambule	bassins	ADJ+NOM+NOM	0,52	0,52	0,52	8	CV-CV-CV-CV-CV-CVC-CV-CV	CV	CV	CV	CV	CV	CVC	CV	CV
bricoleur	invitées	payées	NOM+NOM+ADJ	0,52	0,52	0,52	8	CCV-CV-CVC-V-CV-CV-CV-YV	CCV	CV	CVC	V	CV	CV	CV	YV
assoupi	expressif	coulée	ADJ+NOM+NOM	0,53	0,53	0,53	8	V-CV-CV-VC-CCV-CVC-CV-CV	V	CV	CV	VC	CCCV	CVC	CV	CV
rembourrage	végétale	férié	ADJ+ADJ+ADJ	0,51	0,51	0,51	8	CV-CV-CVC-CV-CV-CVC-CV-CYV	CV	CV	CVC	CV	CV	CVC	CV	CYV
ossature	titulaires	trajets	NOM+NOM+NOM	0,52	0,53	0,51	8	V-CV-CVC-CV-CV-CVC-CCV-CV	V	CV	CVC	CV	CV	CVC	CCV	CV
			**MEAN**	**1,03**												
			**SD**	**0,72**												

**DISTRIBUTION SYLL STRUCT**		N	**%**
V	6	61	**77**
CV	53		
VC	1		
YV	1		

CCV	3		
CVC	13		
CCYV	0		
CYV	2	18	**23**
CYVC	0		
CCVC	0		
CVCC	0		

List of eight-syllable untrained words (B)

Immediate repetition

Word 1	Word 2	Word 3	cgram	freqWord 1	freqWord 2	freqWord 3	nbsyll	cv-cv	S1	S2	S3	S4	S5	S6	S7	S8
méritant	radiologue	navrant	ADJ+NOM+NOM	0,51	0,51	0,51	8	CV-CV-CV-	CV	CV	CV	CV	CYV	CVC	CV	CCV
guillemets	battement	cerceau	NOM+NOM+NOM	0,5	2,59	0,52	8	CV-YV-CV-CV-CV-CV-CVC-CV	CV	YV	CV	CV	CV	CV	CVC	CV
perroquets	anormal	choquée	NOM+NOM+ADJ	1,17	1,19	1,02	8	CV-CV-CV-V-CVC-CVC-CV-CV	CV	CV	CV	V	CVC	CVC	CV	CV
retraité	tromperie	savons	NOM+NOM+NOM	1,17	1,17	1,02	8	CV-CCVC-CV-CCV-CV-CV-CV-CV	CV	CCVC	CV	CCV	CV	CV	CV	CV
érudit	aliment	abîmes	NOM+NOM+NOM	1,09	1,11	0,98	8	V-CV-CV-V-CV-CV-V-CVC	V	CV	CV	V	CV	CV	V	CVC
affilée	facultés	coussins	ADJ+NOM+NOM	2,8	2,8	1,98	8	V-CV-CV-CV-CVC-CV-CV-CV	V	CV	CV	CV	CVC	CV	CV	CV
motivé	pavillons	reçue	ADJ+NOM+ADJ	0,56	0,56	0,55	8	CV-CV-CV-CV-YV-CV-CV-CV	CV	CV	CV	CV	YV	CV	CV	CV
dépendant	étourdi	codée	ADJ+ADJ+ADJ	0,56	0,56	0,55	8	CV-CV-CV-CV-CVC-CV-CV-CV	CV	CV	CV	V	CVC	CV	CV	CV
ordonné	prévoyant	cité	ADJ+ADJ+ADJ	0,57	0,58	0,57	8	VC-CV-CV-CCV-CYV-CYV-YV-CV-CV	VC	CV	CV	CCV	CYV	YV	CV	CV
parfumé	résistant	fumé	ADJ+ADJ+ADJ	0,64	1,77	1,97	8	CVC-CV-CV-CVC-CV-CV-CV	CVC	CV	CV	CV	CVC	CV	CV	CV
			**MEAN**	**1,07**												
			**SD**	**0,71**												

DISTRIBUTION SYLL STRUCT		N	%
V	6	56	**79**
CV	46		
VC	1		
YV	3		
CCV	3	15	**21**
CVC	9		
CCYV	0		
CYV	2		
CYVC	0		
CCVC	1		
YVC	0		
CVCC	0		

Delayed repetition

Word 1	Word 2	Word 3	cgram	freqWord 1	freqWord 2	freqWord 3	nbsyll	cv-cv	S1	S2	S3	S4	S5	S6	S7	S8
insomnie	résolue	troupeaux	NOM+ADJ+NOM	2,55	2,63	1,76	8	V-CVC-CV-CV-CV-CV-CCV-CV	V	CVC	CV	CV	CV	CV	CCV	CV
réchauffé	caribou	brillants	ADJ+NOM+NOM	0,73	0,74	0,68	8	CV-CV-CV-CV-CV-CV-CCV-YV	CV	CV	CV	CV	CV	CV	CCV	YV
cachalot	brocoli	hardie	NOM+NOM+ADJ	0,7	0,69	0,63	8	CV-CV-CV-CCV-CV-CV-CV-CV	CV	CV	CV	CCV	CV	CV	CV	CV
écorchure	occupées	bavardes	NOM+ADJ+ADJ	0,61	0,61	0,61	8	V-CVC-CVC-V-CV-CV-CV-CVCC	V	CVC	CVC	V	CV	CV	CV	CVCC
écarté	verrouillée	déçu	ADJ+ADJ+NOM	0,6	0,6	0,85	8	V-CVC-CV-CV-CV-YV-CV-CV	V	CVC	CV	CV	CV	YV	CV	CV
éveillé	assurée	lenteur	ADJ+ADJ+NOM	1,39	1,45	1,15	8	V-CV-YV-V-CV-CV-CV-CVC	V	CV	YV	V	CV	CV	CV	CVC
amateurs	odorat	donné	ADJ+NOM+NOM	1,27	1,28	1,19	8	V-CV-CVC-V-CV-CV-CV-CV	V	CV	CVC	V	CV	CV	CV	CV

financières	accusé	citron	ADJ+ADJ+ADJ	1,36	1,37	1,14	8	CV-CV-CYVC-V-CV-CV-CV-CCV	CV	CV	CYVC	V	CV	CV	CV	CCV
délicats	immobiles	averse	ADJ+ADJ+NOM	1,35	1,35	1,15	8	CV-CV-CV-V-CV-CVC-V-CVCC	CV	CV	CV	V	CV	CVC	V	CVCC
allumé	vitamine	fourneaux	ADJ+NOM+NOM	1,3	1,31	1,13	8	V-CV-CV-CV-CV-CVC-CVC-CV	V	CV	CV	CV	CV	CVC	CVC	CV
			**MEAN**	**1,14**												
			**SD**	**0,52**												

**DISTRIBUTION SYLL STRUCT**		N	**%**
V	12	64	**80**
CV	49		
VC	0		
YV	3		
CCV	4	16	**20**
CVC	9		
CCYV	0		
CYV	0		
CYVC	1		
CCVC	0		
CVCC	2		

2. List of trained and untrained pseudowords

List of two-syllable trained pseudowords (A)

Immediate repetition

Word	cv-cv	S1	S2
lerou	CV-CV	CV	CV
kifière	CV-CYVC	CV	CYVC
trampeau	CCV-CV	CCV	CV
vajet	V-CV	V	CV
voinré	CYV-CV	CYV	CV
chuyan	CV-YV	CV	YV
tichet	CV-CV	CV	CV
gyvion	CV-CYVC	CV	CYVC
moupin	CV-CV	CV	CV
sâré	CV-CV	CV	CV

DISTRIBUTION SYLL STRUCT		N	%
V	1	16	**82%**
CV	14		
VC	0		
YV	1		
CCV	1	3	**18%**
CVC	0		
CCYV	0		
CYV	0		
CYVC	2		

Delayed repetition

Word	cv-cv	S1	S2
écroi	V-CCYV	V	CCYV
naron	CV-CV	CV	CV
pouloute	CV-CVC	CV	CVC
paguore	CV-CVC	CV	CVC
urtal	VC-CVC	VC	CVC
fêpu	CV-CV	CV	CV
miber	CV-CV	CV	CV
popu	CV-CV	CV	CV
diman	CV-CV	CV	CV
chozé	CV-CV	CV	CV

DISTRIBUTION SYLL STRUCT		N	%

V	1	16	**80**
CV	14		
VC	1		
YV	0		
CCV	0	4	**20**
CVC	3		
CCYV	1		
CYV	0		
CYVC	0		

List of two-syllable untrained pseudowords (B)

Immediate repetition

Word	cv-cv	S1	S2
tuificre	CYV-CVCC	CYV	CVCC
bero	CV-CV	CV	CV
soru	CV-CV	CV	CV
pouflète	CV-CCVC	CV	CCVC
dorire	CV-CVC	CV	CVC
léante	CV-VC	CV	VC
nafé	CV-CV	CV	CV
guèta	CV-CV	CV	CV
nolu	CV-CV	CV	CV
choumon	CV-CV	CV	CV

DISTRIBUTION SYLL STRUCT		N	%
V	0	16	**80**
CV	15		
VC	1		
YV	0		
CCV	0	4	**20**
CVC	1		
CCYV	0		
CCVC	1		
CYVC	0		
CVCC	1		
CYV	1		

Delayed repetition

Word	cv-cv	S1	S2
napin	CV-CV	CV	CV
tarou	CV-CV	CV	CV

mélin	CV-CV	CV	CV
goza	CV-CV	CV	CV
cluteau	CCV-CV	CCV	CV
soinchain	CYV-CV	CYV	CV
pachu	CV-CV	CV	CV
rouflette	CV-CCVC	CV	CCVC
berin	CV-CV	CV	CV
bagueuil	CV-CVY	CV	CVY

DISTRIBUTION SYLL STRUCT		N	%
V	0	16	**80**
CV	16		
VC	0		
YV	0		
CCV	1	4	**20**
CVC	0		
CVY	1		
CYV	1		
CYVC	0		
CCVC	1		

List of three-syllable trained pseudowords (A)

Immediate repetition

Word	cv-cv	S1	S2	S3
pacubaire	CV-CV-CVC	CV	CV	CVC
tadékour	CVC-CV-CVC	CVC	CV	CVC
chamago	CV-CV-CV	CV	CV	CV
ouchoki	VC-CV-CV	VC	CV	CV
afuyé	V-CYV-YV	V	CYV	YV
namycaire	VC-CV-CVC	VC	CV	CVC
atimeau	V-CV-CV	V	CV	CV
entanké	V-CV-CV	V	CV	CV
arlipo	VC-CV-CV	VC	CV	CV
oubrica	V-CCV-CV	V	CCV	CV

DISTRIBUTION SYLL STRUCT		N	%
V	4	24	**80**
CV	16		
VC	3		
YV	1		
CCV	1	6	**20**

CVC	4		
CCYV	0		
CYV	1		
CYVC	0		

Delayed repetition

Word	cv-cv	S1	S2	S3
fesiron	CV-CV-CV	CV	CV	CV
hautoyé	V-CYV-YV	V	CYV	YV
tirépli	V-CV-CCV	V	CV	CCV
carindor	CVC-CV-CV	CVC	CV	CVC
bimanko	CV-CV-CV	CV	CV	CV
roupernelle	CV-CVC-CVC	CV	CVC	CVC
poumouto	CV-CV-CV	CV	CV	CV
imotan	V-CV-CV	V	CV	CV
ensira	V-CV-CV	V	CV	CV
opazé	V-CV-CV	V	CV	CV

DISTRIBUTION SYLL STRUCT		N	%
V	5	23	**79%**
CV	17		
VC	0		
YV	1		
CCV	1	6	**21%**
CVC	4		
CCYV	0		
CYV	1		
CYVC	0		

List of three-syllable untrained pseudowords (B)

Immediate repetition

Word	cv-cv	S1	S2	S3
rédichon	CV-CV-CV	CV	CV	CV
mofadin	CV-CV-CV	CV	CV	CV
ilomable	V-CV-CVCC	V	CV	CVCC
poumataille	CV-CV-CVY	CV	CV	CVY
oulcrapon	VC-CCV-CV	VC	CCV	CV
moverpaille	CV-CVC-CVY	CV	CVC	CVY
beremen	CV-CV-CV	CV	CV	CV
amplyon	V-CCV-YV	V	CCV	YV
tormoulé	CVC-CV-CV	CVC	CV	CV

chobeba	V-CV-CV	V	CV	CV

DISTRIBUTION SYLL STRUCT		N	%
V	3	23	**77%**
CV	18		
VC	1		
YV	1		
CCV	2	7	**23%**
CVC	2		
CCYV	0		
CYV	0		
CVY	2		
CVCC	1		

Delayed repetition

Word	cv-cv	S1	S2	S3
agravyon	V-CCVC-CYV	V	CCVC	CYV
phifrény	CV-CV-CV	CV	CV	CV
enlouné	V-CV-CV	V	CV	CV
taillechan	CV-YV-CV	CV	YV	CV
mouglichan	CV-CCV-CV	CV	CCV	CV
étolan	V-CV-CV	V	CV	CV
sétaverle	CV-CV-CVCC	CV	CV	CVCC
péranda	CV-CV-CV	CV	CV	CV
ritélaire	CV-CV-CVC	CV	CV	CVC
pakitaje	CV-CV-CVC	CV	CV	CVC

DISTRIBUTION SYLL STRUCT		N	%
V	3	24	**80**
CV	20		
VC	0		
YV	1		
CCV	1	6	**20**
CVCC	1		
CVC	2		
CYV	1		
CYVC	0		
CCVC	1		

List of four-syllable trained pseudowords (A)

Immediate repetition

Word	cv-cv	S1	S2	S3	S4
dalitaban	CV-CV-CV-CV	CV	CV	CV	CV
tuplirimé	CV-CCV-CV-CV	CV	CCV	CV	CV
pérutricaine	CV-CV-CCV-CVC	CV	CV	CCV	CVC
nunalépie	CV-CV-CV-CV	CV	CV	CV	CV
lépaniré	CV-CV-CV-CV	CV	CV	CV	CV
foncorjité	CV-CVC-CV-CV	CV	CVC	CV	CV
ingranchassion	V-CCV-CV-CYV	V	CCV	CV	CYV
ipuchandu	V-CV-CV-CV	V	CV	CV	CV
inlesucreur	V-CV-CVC-CVC	V	CV	CVC	CVC
galaboucrabe	CV-CV-CVC-CV	CV	CV	CVC	CV

DISTRIBUTION SYLL STRUCT		N	%
V	3	24	**80**
CV	21		
VC	0		
YV	0		
CCV	3	6	**20**
CVC	3		
CCYV	0		
CYV	0		
CYVC	0		

Delayed repetition

Word	cv-cv	S1	S2	S3	S4
cinoparlé	CV-CV-CVC-CV	CV	CV	CVC	CV
rédélonatre	CV-CV-CV-CVCC	CV	CV	CV	CVCC
fidodémou	CV-CV-CV-CV	CV	CV	CV	CV
filurtikal	CV-CVC-CV-CVC	CV	CVC	CV	CVC
mielpaniroi	CYV-CYV-CV-CV	CYV	CYV	CV	CV
hébeaunobar	V-CV-CV-CVC	V	CV	CV	CVC
breyoulaman	CCV-YV-CV-CV	CCV	YV	CV	CV
brossachement	CCV-CV-CV-CV	CCV	CV	CV	CV
tréfoulopi	CCV-CV-CV-CV	CCV	CV	CV	CV
engomalée	V-CV-CV-CV	V	CV	CV	CV

DISTRIBUTION SYLL STRUCT		N	%
V	2	23	**77**
CV	20		
VC	0		
YV	1		

CCV	3	7	**23**
CVC	2		
CCYV	0		
CYV	2		
CYVC	0		

List of four-syllable untrained pseudowords (B)

Immediate repetition

Word	cv-cv	S1	S2	S3	S4
trolipéfiée	CCV-CV-CV-CYV	CCV	CV	CV	CYV
impauréssion	V-CV-CV-CYV	V	CV	CV	CYV
saporinou	V-CV-CV-CV	V	CV	CV	CV
nagnumara	CVC-CV-CV-CV	CVC	CV	CV	CV
timitoulet	CV-CV-CV-CV	CV	CV	CV	CV
anmareutée	V-CV-CV-CV	V	CV	CV	CV
inturproniale	V-CVC-CCV-YVC	V	CVC	CCV	YVC
rasifichet	V-CV-CV-CV	V	CV	CV	CV
fongolopinque	CV-CV-CV-CVC	CV	CV	CV	CVC
otalimo	V-CV-CV-CV	V	CV	CV	CV

**DISTRIBUTION SYLL STRUCT**		N	**%**
V	6	32	**80**
CV	26		
VC	0		
YV	0		
CCV	2	8	**20**
CVC	3		
CCYV	0		
CYV	2		
CYVC	0		
YVC	1		

Delayed repetition

Word	cv-cv	S1	S2	S3	S4
chalafoutan	CV-CV-CV-CV	CV	CV	CV	CV
ségagrionnaire	CV-VC-CYV-CVC	CV	VC	CYV	CVC
amarafie	V-CV-CV-CV	V	CV	CV	CV

inlongrapable	V-CV-CCV-CVCC	V	CV	CCV	CVCC
antoréchet	V-CV-CV-CV	V	CV	CV	CV
agocarpie	V-CV-CVC-CV	V	CV	CVC	CV
aplambereau	VC-CCV-CV-CV	V	CCV	CV	CV
lafatimé	CV-CV-CV-CV	CV	CV	CV	CV
apéchement	V-CV-CV-CV	V	CV	CV	CV
imbontarnagle	V-CV-CVC-CVCC	V	CV	CVC	CVCC

DISTRIBUTION SYLL STRUCT		N	%
V	7	32	**80**
CV	24		
VC	1		
YV	0		
CCV	2	8	**20**
CVC	3		
CCYV	0		
CYV	1		
CYVC	0		
YVC	0		
CVCC	2		

List of five-syllable trained pseudowords (A)

Immediate repetition

Word	cv-cv	S1	S2	S3	S4	S5
archirénétan	VC-CV-CV-CV-CV	VC	CV	CV	CV	CV
intervaliorame	V-CVC-CV-CYV-CVC	V	CVC	CV	CYV	CVC
pélonchorission	CV-CV-CV-CV-CYV	CV	CV	CV	CV	CYV
teulapimanko	CV-CV-CV-CV-CV	CV	CV	CV	CV	CV
glaritérifian	CCV-CV-CV-CV-CV	CCV	CV	CV	CV	CV
fatotubaka	CV-CV-CV-CV-CV	CV	CV	CV	CV	CV
métopajalion	CV-CV-CV-CV-CYV	CV	CV	CV	CV	CYV
arvéolachique	VC-CV-V-CV-CVC	VC	CV	V	CV	CVC
nupariotilé	CV-CV-CYV-CV-CV	CV	CV	CYV	CV	CV
olitêbéssan	V-CV-CV-CV-CV	V	CV	CV	CV	CV

DISTRIBUTION SYLL STRUCT		N	%
V	3	42	**84**
CV	37		
VC	2		
YV	0		

CCV	1	8	**16**
CVC	3		
CYV	4		

Delayed repetition

Word	cv-cv	S1	S2	S3	S4	S5
épilieulotan	V-CV-CYV-CV-CV	V	CV	CYV	CV	CV
oligaliman	V-CV-CV-CV-CV	V	CV	CV	CV	CV
dolpivassionèle	CVC-CV-CV-CYV-CVC	CVC	CV	CV	CYV	CVC
prédichétassiou	CCV-CV-CV-CV-CYV	CCV	CV	CV	CV	CYV
indovapaman	V-CV-CV-CV-CV	V	CV	CV	CV	CV
choupitaillement	CV-CV-CV-YV-CV	CV	CV	CV	YV	CYV
lutafanrassion	CV-CV-CV-CV-CYV	CV	CV	CV	CV	CYV
coardinacheur	CV-VC-CV-CV-CVC	CV	VC	CV	CV	CVC
imméralifé	V-CV-CV-CV-CV	V	CV	CV	CV	CV
féguloritou	CV-CV-CV-CV-CV	CV	CV	CV	CV	CV

DISTRIBUTION SYLL STRUCT		N	%
V	4	41	**82**
CV	35		
VC	1		
YV	1		
CCV	1	9	**18**
CVC	3		
CYV	5		

List of five-syllable untrained pseudowords (B)

Immediate repetition

Word	cv-cv	S1	S2	S3	S4	S5
rolémoratiche	CV-CV-CV-CV-CVC	CV	CV	CV	CV	CVC
impélenchaman	V-CV-CV-CV-CV	V	CV	CV	CV	CV
fanjeroucheman	CV-CV-CV-CV-CV	CV	CV	CV	CV	CV
orbidulassion	VC-CV-CV-CV-CYV	VC	CV	CV	CV	CYV
interblanéfoure	V-CVC-CCV-CV-CVC	V	CVC	CCV	CV	CVC
gamacrélistique	CV-CVC-CV-CVC-CVC	CV	CVC	CV	CVC	CVC
arétissevan	V-CV-CV-CV-CV	V	CV	CV	CV	CV
méalifatrisse	CV-V-CV-CV-CCVC	CV	V	CV	CV	CCVC
rumilasité	CV-CV-CV-CV-CV	CV	CV	CV	CV	CV
orfanilassion	VC-CV-CV-CV-CYV	VC	CV	CV	CV	CYV

DISTRIBUTION SYLL STRUCT		N	%
V	4	40	**80**
CV	34		
VC	2		
YV	0		
CCV	1	10	**20**
CVC	6		
CYV	2		
CCVC	1		

Delayed repetition

Word	cv-cv	S1	S2	S3	S4	S5
orinassinoble	V-CV-CV-CV-CVCC	V	CV	CV	CV	CVCC
talifigateur	CV-CV-CV-CV-CVC	CV	CV	CV	CV	CV
clouchiricassion	CCV-CV-CV-CV-CYV	CCV	CV	CV	CV	CYV
orégimassion	V-CV-CV-CV-CYV	V	CV	CV	CV	CYV
amertilévan	V-CVC-CV-CV-CV	V	CVC	CV	CV	CV
karipoussateur	CV-CV-CV-CV-CVC	CV	CV	CV	CV	CVC
lectiricassion	CVC-CV-CV-CV-CYV	CVC	CV	CV	CV	CYV
magatirité	CV-CV-CV-CV-CV	CV	CV	CV	CV	CV
ouchapijité	V-CV-CV-CV-CV	V	CV	CV	CV	CV
imperrogalioure	V-CV-CV-CV-CYVC	V	CV	CV	CV	CYVC

DISTRIBUTION SYLL STRUCT		N	%
V	5	41	**82**
CV	36		
VC	0		
YV	0		
CCV	1	9	**18**
CVC	3		
CYV	3		
CVCC	1		
CYVC	1		

List of six-syllable trained pseudowords (A)

Immediate repetition

Word 1	Word 2	cv-cv	S1	S2	S3	S4	S5	S6
unila	gardosan	V-CV-CV-CVC-CV-CV	V	CV	CV	CVC	CV	CV
gridijère	teaumiré	CCV-CV-CVC-CV-CV-CV	CCV	CV	CVC	CV	CV	CV
roquélan	poularil	CV-V-CV-CV-CVC-CV	CV	V	CV	CV	CVC	CV
apomieau	urité	V-CV-CYV-V-CV-CV	V	CV	CYV	V	CV	CV

clapadil	lanbassé	CCV-CV-CVC-CV-CV-CV	CCV	CV	CVC	CV	CV	CV
patrier	lofaïque	CV-CCV-YV-CV-CV-VC	CV	CCV	YV	CV	CV	VC
intanveau	séguidé	V-CV-CV-CV-CV-CV	V	CV	CV	CV	CV	CV
rivougué	entanlire	CV-CV-CV-V-CV-CVC	CV	CV	CV	V	CV	CVC
anmordi	sipraivon	V-CVC-CV-CV-CCV-CV	V	CVC	CV	CV	CCV	CV
rapental	entoulu	CV-CV-CVC-V-CV-CV	CV	CV	CVC	V	CV	CV

DISTRIBUTION SYLL STRUCT		N	%
V	8	48	**80**
CV	38		
VC	1		
YV	1		
CCV	4	12	**20**
CVC	7		
CCYV	0		
CYV	1		
CYVC	0		

Delayed repetition

Word 1	Word 2	cv-cv	S1	S2	S3	S4	S5	S6
ponfinan	taudrénan	CV-CV-CV-CV-CCV-CV	CV	CV	CV	CV	CCV	CV
factrition	tomelette	CVC-CCVC-CYV-CV-CV-CVC	CVC	CCVC	CYV	CV	CV	CVC
iratif	rabaseau	V-CV-CVC-CV-CV-CV	V	CV	CVC	CV	CV	CV
galagnion	néputia	CV-CV-CYV-CV-CV-CYV	CV	CV	CYV	CV	CV	CYV
annobé	artabète	V-CV-CV-VC-CV-CVC	V	CV	CV	VC	CV	CVC
acharé	tonoubeu	V-CV-CV-CV-CV-CV	V	CV	CV	CV	CV	CV
florasie	nouméגon	CCV-CV-CV-CV-CV-CV	CCV	CV	CV	CV	CV	CV
ammépire	redeumé	VC-CV-CVC-CV-CV-CV	VC	CV	CVC	CV	CV	CV
antipa	sidonaure	V-CV-CV-CV-CV-CVC	V	CV	CV	CV	CV	CVC
béruchin	romenet	CV-CV-CV-CV-CV-CV	CV	CV	CV	CV	CV	CV

DISTRIBUTION SYLL STRUCT		N	%
V	4	48	**81%**
CV	42		
VC	2		
YV	0		
CCV	2	11	**19%**
CVC	6		
CCYV	0		
CYV	3		
CYVC	0		

List of six-syllable untrained pseudowords (B)

Immediate repetition

Word 1	Word 2	cv-cv	S1	S2	S3	S4	S5	S6
inlintu	moteulier	V-CV-CV-CV-CV-CYV	V	CV	CV	CV	CV	CYV
nadékan	raronchante	CV-CV-CV-CV-CV-CVC	CV	CV	CV	CV	CV	CVC
daliation	élotier	CV-CYV-CYV-V-CV-CYV	CV	CYV	CYV	V	CV	CYV
dymophée	almanie	CV-CV-CV-VC-CV-CV	CV	CV	CV	VC	CV	CV
astrapneau	répenjambe	VC-CV-CCVC-CV-CV-CVC	VC	CV	CCVC	CV	CV	CVC
ingréfi	onuipette	V-CCV-CV-V-CYV-CVC	V	CCV	CV	V	CYV	CVC
piméloure	atendeur	CV-CV-CVC-V-CV-CVC	CV	CV	CVC	V	CV	CVC
tégriment	touferin	CV-CCV-CV-CV-CV-CV	CV	CCV	CV	CV	CV	CV
assoteau	charémal	V-CV-CV-CV-CV-CVC	V	CV	CV	CV	CV	CVC
tolaneur	uliton	CV-CV-CVC-CV-CV-CV	CV	CV	CV	CV	CV	CV

DISTRIBUTION SYLL STRUCT		N	%
V	6	46	**77%**
CV	38		
VC	2		
YV	0		
CCV	2	14	**23%**
CVC	6		
CCYV	0		
CYV	5		
CVY	0		
CCVC	1		

Delayed repetition

Word 1	Word 2	cv-cv	S1	S2	S3	S4	S5	S6
pracaril	yeséca	CCV-CV-CVC-CV-CV-CV	CCV	CV	CVC	CV	CV	CV
fanijeur	marobètre	CV-CV-CVC-CV-CV-CVCC	CV	CV	CVC	CV	CV	CVCC
aproumeau	inloprier	V-CV-CV-V-CVC-CYV	V	CV	CV	V	CVC	CYV
thifimi	misagrée	CV-CV-CV-CV-CV-CCV	CV	CV	CV	CV	CV	CCV
toinfilleux	entanpin	CYV-CV-YV-V-CV-CV	CYV	CV	YV	V	CV	CV
itumé	boncubire	V-CV-CV-CV-CV-CVC	V	CV	CV	CV	CV	CVC
narjidage	danronée	CVC-CV-CVC-CV-CV-CV	CVC	V	CVC	CV	CV	CV
asécreur	rutelé	V-CVC-CVC-CV-CV-CV	V	CVC	CVC	CV	CV	CV
nulafique	limirètre	CV-CV-CVC-CV-CV-CVCC	CV	CV	CVC	CV	CV	CVCC
drobelie	jiditeaule	CCV-CV-CV-CV-CV-CVC	CCV	CV	CV	CV	CV	CVC

DISTRIBUTION SYLL STRUCT		N	%
V	6	42	**76%**

CV	35		
VC	0		
YV	1		
CCV	3	13	**23%**
CVCC	2		
CVC	6		
CYV	2		
CYVC	0		
CCVC	0		

List of seven-syllable trained pseudowords (A)

Immediate repetition

Word 1	Word 2	Word 3	cv-cv	S1	S2	S3	S4	S5	S6	S7
zibon	qualet	latiment	CV-CV-CV-CV-CV-CV-CV	CV	CV	CV	CV	CV	CV	CV
lopour	rajeau	inquidet	CV-CVC-CV-CV-V-CV-CV	CV	CVC	CV	CV	V	CV	CV
joreau	tenlille	rinemai	CV-CV-CV-CVY-CV-CV-CV	CV	CV	CV	CVY	CV	CV	CV
loubeau	tremeur	labanssé	CV-CV-CCV-CVC-CV-CV-CV	CV	CV	CCV	CVC	CV	CV	CV
atance	houlain	sotignol	V-CVC-V-CV-CV-CV-CVC	V	CVC	V	CV	CV	CV	CVC
rafeau	trocher	donferie	CV-CV-CCV-CV-CV-CV-CV	CV	CV	CCV	CV	CV	CV	CV
teillé	uchée	raliation	CV-YV-V-CV-CV-CYV-CYV	CV	YV	V	CV	CV	CYV	CYV
tempeur	burtine	péranlu	CV-CVC-CVC-CVC-CV-CV-CV	CV	CVC	CVC	CVC	CV	CV	CV
bofie	épeau	calcachour	CV-CV-V-CV-CVC-CV-CVC	CV	CV	V	CV	CVC	CV	CVC
lofant	rachaine	litilante	CV-CV-CV-CVC-CV-CV-CVC	CV	CV	CV	CVC	CV	CV	CVC

DISTRIBUTION SYLL STRUCT		N	%
V	5	54	**78**
CV	48		
VC	0		
YV	1		
CCV	2	15	**22**
CVC	11		
CCYV	0		
CYV	2		
CYVC	0		

CCVC	0		
YVC	0		
CVCC	0		

Delayed repetition

Word 1	Word 2	Word 3	cv-cv	S1	S2	S3	S4	S5	S6	S7
poutole	vidin	ratahon	CV-CVC-CV-CV-CV-CV-V	CV	CVC	CV	CV	CV	CV	V
taran	chointu	inmenché	CV-CV-CYV-CV-V-CV-CV	CV	CV	CYV	CV	V	CV	CV
pulite	lomet	rylanan	CV-CVC-CV-CV-CV-CV-CV	CV	CVC	CV	CV	CV	CV	CV
renlé	létompre	maillaton	CV-CV-CVCC-CV-YV-CV	CV	CV	CV	CVCC	CV	YV	CV
réffée	mosset	lakorie	CV-CV-CV-CV-CV-CV-CV	CV	CV	CV	CV	CV	CV	CV
tabu	torchune	indréfient	CV-CV-CVC-CVC-V-CCV-CYV	CV	CV	CVC	CVC	V	CCV	CYV
itard	maichiou	anlifope	V-CVC-CV-CYV-V-CV-CVC	V	CVC	CV	CYV	V	CV	CVC
toré	fenchu	tesanpeur	CV-CV-CV-CV-CV-CV-CVC	CV	CV	CV	CV	CV	CV	CVC
graseau	sitile	rédanchée	CCV-CV-CV-CVC-CV-CV-CV	CCV	CV	CV	CVC	CV	CV	CV
choutée	répon	lédégueau	CV-CV-CV-CV-CV-CV-CV-CV	CV	CV	CV	CV	CV	CV	CV

DISTRIBUTION SYLL STRUCT		N	%
V	5	56	**83%**
CV	50		
VC	0		
YV	1		
CCV	2	11	**16%**
CVC	8		
CCYV	0		
CYV	0		
CYVC	0		
CCVC	0		
CVCC	1		

List of seven-syllable untrained pseudowords (B)

Immediate repetition

Word 1	Word 2	Word 3	cv-cv	S1	S2	S3	S4	S5	S6	S7

tilornes	alchin	tonsenpant	CV-CVCC-VC-CV-CV-CV-CV	CV	CVCC	VC	CV	CV	CV	CV
rulin	milleul	échanouie	CV-CV-CV-YVC-V-CV-CYV	CV	CV	CV	YVC	V	CV	CYV
grouleau	pachon	inchivé	CCV-CV-CV-CV-V-CV-CV	CCV	CV	CV	CV	V	CV	CV
rignés	serlant	ajourti	CVC-CV-CV-CV-V-CVC-CV	CVC	CV	CV	CV	V	CVC	CV
lyrvuose	aqui	marnegant	CVC-CYVC-V-CV-CVC-CV-CV	CVC	CYVC	V	CV	CVC	CV	CV
némage	popou	vonrainlant	CV-CVC-CV-CV-CV-CV-CV	CV	CVC	CV	CV	CV	CV	CV
tonquis	donlu	nourjalier	CV-CV-CV-CV-CVC-CV-CYV	CV	CV	CV	CV	CVC	CV	CYV
toulé	péki	intanchée	CV-CV-CV-CV-V-CV-CV	CV	CV	CV	CV	V	CV	CV
dalé	ousseau	lirantesque	CV-CV-V-CV-CV-CV-CVCC	CV	CV	V	CV	CV	CV	CVCC
leuteux	chédet	bonlateur	CV-CV-CV-CV-CV-CV-CVC	CV	CV	CV	CV	CV	CV	CVC

DISTRIBUTION SYLL STRUCT		N	%
V	5	56	**80**
CV	49		
VC	2		
YV	0		
CCV	1	14	**20**
CVC	7		
CCYV	0		
CYV	2		
CYVC	1		
CCVC	0		
YVC	1		
CVCC	2		

Delayed repetition

Word 1	Word 2	Word 3	cv-cv	S1	S2	S3	S4	S5	S6	S7
fachin	moupli	mantolant	CV-CV-CV-CCV-CV-CV-CV	CV	CV	CV	CCV	CV	CV	CV
balère	uset	lonjeré	CV-CVC-V-CV-CV-CV-CV	CV	CVC	V	CV	CV	CV	CV
vaca	choubé	natiloire	CV-CVC-V-CV-CV-CV-CYVC	CV	CV	CV	CV	CV	CV	CYVC
merfée	pinron	fréséjie	CV-CV-CVC-CV-CCV-CV-CV	CV	CV	CVC	CV	CCV	CV	CV
merlite	abtan	naradien	CVC-CVC-VC-CV-CV-CV-CYV	CVC	CVC	VC	CV	CV	CV	CYV
sada	litan	frapmuret	CV-CV-CV-CV-CCVC-CV-CV	CV	CV	CV	CV	CCVC	CV	CV
luret	loureau	chubelon	CV-CV-CV-CV-CV-CV-CV	CV	CV	CV	CV	CV	CV	CV

rujiste	tafé	lopontaire	CV-CVCC-CV-CV-CV-CV-CVC	CV	CVCC	CV	CV	CV	CV	CVC
jizette	dingué	tépillant	CV-CVC-CV-CV-CV-CV-YV	CV	CVC	CV	CV	CV	CV	YV
clillée	tonman	déliévale	CCV-YV-CV-CV-CV-CYV-CVC	CCV	YV	CV	CV	CV	CYV	CVC

DISTRIBUTION SYLL STRUCT		N	%
V	1	55	**80%**
CV	51		
VC	1		
YV	2		
CCV	3	14	**20%**
CVC	7		
CCYV	0		
CYV	1		
CYVC	1		
CCVC	1		
CVCC	1		

List of eight-syllable trained pseudowords (A)

Immediate repetition

Word 1	Word 2	Word 3	cv-cv	S1	S2	S3	S4	S5	S6	S7	S8
tasamé	traferneige	oli	CV-CV-CV-CCV-CVC-CVC-V-CV	CV	CV	CV	CCV	CVC	CVC	V	CV
lorsifelle	rifétan	adrielle	CVC-CV-CVC-CV-CV-CV-VC-CYVC	CVC	CV	CVC	CV	CV	CV	VC	CYVC
fazinée	hopichon	tichale	CV-CV-CV-CV-CV-CV-CV-CVC	CV	CV	CV	V	CV	CV	CV	CVC
salouitance	arata	jané	CV-CYV-CVC-V-CV-CV-CV-CV	CV	CYV	CVC	V	CV	CV	CV	CV
clanton	damené	monflé	CV-CV-CV-CV-CV-CV-CV-CCV	CCV	CV	CV	CV	CV	CV	CV	CCV
lambéchan	moqunal	milon	CV-CV-CV-CV-CV-CVC-CV-CV	CV	CV	CV	CV	CV	CVC	CV	CV
envurli	apenant	toumis	V-CVC-CV-V-CV-CV-CV-CV	V	CVC	CV	V	CV	CV	CV	CV
aritant	éclayée	lantée	V-CCV-YV-CV-CV-CV-CV-CV	V	CCV	YV	CV	CV	CV	CV	CV
ilpomiste	amineau	fandou	VC-CV-CVCC-V-CV-CV-CV-CV	VC	CV	CVCC	V	CV	CV	CV	CV
saradé	fondérant	pasé	CV-CV-CV-CV-CV-CV-CV-CV	CV	CV	CV	CV	CV	CV	CV	CV

DISTRIBUTION SYLL STRUCT		N	%
V	7	56	**80%**
CV	46		
VC	2		
YV	1		

CCV	4	15	**20%**
CVC	8		
CCYV	0		
CYV	1		
CYVC	1		
CCVC	0		
YVC	0		
CVCC	1		

Delayed repetition

Word 1	Word 2	Word 3	cv-cv	S1	S2	S3	S4	S5	S6	S7	S8
éfordé	terinon	saret	V-CVC-CV-CV-CV-CV-CV-CV	V	CVC	CV	CV	CV	CV	CV	CV
sidoreau	lérenjeur	ampeux	CV-CV-CV-CV-CV-CVC-V-CV	CV	CV	CV	CV	CV	CVC	V	CV
vonrainlu	tronjement	lorteuse	CV-CV-CV-CCV-CV-CV-CV-CV	CV	CV	CV	CCV	CV	CV	CVC	CVC
tumilé	tomacal	poret	CV-CV-CV-CV-CV-CVC-CV-CV	CV	CV	CV	CV	CV	CVC	CV	CV
achilé	toquidien	lijide	V-CV-CV-CV-CYV-CV-CVC	V	CV	CV	CV	CV	CYV	CV	CVC
léchobée	tunambale	talin	CV-CV-CV-CV-CV-CVC-CV-CV	CV	CV	CV	CV	CV	CVC	CV	CV
trigoleur	impivée	layée	CCV-CV-CVC-V-CV-CV-CV-YV	CCV	CV	CVC	V	CV	CV	CV	YV
apouchi	extressil	mouvée	V-CV-CV-VC-CCV-CVC-CV-CV	V	CV	CV	VC	CCCV	CVC	CV	CV
lantapage	téjévale	réfié	CV-CV-CVC-CV-CV-CVC-CV-CYV	CV	CV	CVC	CV	CV	CVC	CV	CYV
atarure	vipulaire	crateau	V-CV-CVC-CV-CV-CVC-CCV-CV	V	CV	CVC	CV	CV	CVC	CCV	CV

DISTRIBUTION SYLL STRUCT		N	%
V	6	61	**77%**
CV	53		
VC	1		
YV	1		
CCV	3	18	**23%**
CVC	13		
CCYV	0		
CYV	2		
CYVC	0		
CCVC	0		
CVCC	0		

List of eight-syllable untrained pseudowords (B)

Immediate repetition

Word 1	Word 2	Word 3	cv-cv	S1	S2	S3	S4	S5	S6	S7	S8
térimant	paliologue	vagrant	CV-CV-CV-	CV	CV	CV	CV	CYV	CVC	CV	CCV
tuillemeau	poulement	mermeau	CV-YV-CV-CV-CV-CV-CVC-CV	CV	YV	CV	CV	CV	CV	CVC	CV
répoquet	ilarmèle	tové	CV-CV-CV-V-CVC-CVC-CV-CV	CV	CV	CV	V	CVC	CVC	CV	CV
legraipet	fromperie	vaton	CV-CCVC-CV-CCV-CV-CV-CV-CV	CV	CCVC	CV	CCV	CV	CV	CV	CV
édurie	oupiment	afîme	V-CV-CV-V-CV-CV-V-CVC	V	CV	CV	V	CV	CV	V	CVC
aliffée	cafulté	moussin	V-CV-CV-CV-CVC-CV-CV-CV	V	CV	CV	CV	CVC	CV	CV	CV
tomivé	vatillon	sauru	CV-CV-CV-CV-YV-CV-CV-CV	CV	CV	CV	CV	YV	CV	CV	CV
léfendant	élourmi	poudée	CV-CV-CV-CV-CVC-CV-CV-CV	CV	CV	CV	V	CVC	CV	CV	CV
arlonnée	préchoyant	fipeau	VC-CV-CV-CCV-CYV-CYV-YV-CV-CV	VC	CV	CV	CCV	CYV	YV	CV	CV
farchumet	tésicrant	mufé	CVC-CV-CV-CVC-CV-CV-CV	CVC	CV	CV	CV	CVC	CV	CV	CV

DISTRIBUTION SYLL STRUCT		N	%
V	6	56	**79%**
CV	46		
VC	1		
YV	3		
CCV	3	15	**21%**
CVC	9		
CCYV	0		
CYV	2		
CYVC	0		
CCVC	1		
YVC	0		
CVCC	0		

Delayed repetition

Word 1	Word 2	Word 3	cv-cv	S1	S2	S3	S4	S5	S6	S7	S8
impagrie	lérozu	trapeau	V-CVC-CV-CV-CV-CV-CCV-CV	V	CVC	CV	CV	CV	CV	CCV	CV
pérauffé	baricou	trillant	CV-CV-CV-CV-CV-CV-CCV-YV	CV	CV	CV	CV	CV	CV	CCV	YV
mapoulot	tropoli	ildeau	CV-CV-CV-CCV-CV-CV-CV-CV	CV	CV	CV	CCV	CV	CV	CV	CV
étormur	occuffée	vabarde	V-CVC-CVC-V-CV-CV-CV-CVCC	V	CVC	CVC	V	CV	CV	CV	CVCC
élarté	merraillée	tenleur	V-CVC-CV-CV-CV-YV-CV-CV	V	CVC	CV	CV	CV	YV	CV	CV
égaillé	amurée	venteur	V-CV-YV-V-CV-CV-CV-CVC	V	CV	YV	V	CV	CV	CV	CVC
atameur	amora	lonné	V-CV-CVC-V-CV-CV-CV-CV	V	CV	CVC	V	CV	CV	CV	CV
rinantière	afusé	ligron	CV-CV-CYVC-V-CV-CV-CV-CCV	CV	CV	CYVC	V	CV	CV	CV	CCV

mélicha	iffobile	aterse	CV-CV-CV-V-CV-CVC-V-CVCC	CV	CV	CV	V	CV	CVC	V	CVCC
apumé	tivaline	toulreaux	V-CV-CV-CV-CV-CVC-CVC-CV	V	CV	CV	CV	CV	CVC	CVC	CV

**DISTRIBUTION SYLL STRUCT**		N	%
V	12	64	**80%**
CV	49		
VC	0		
YV	3		
CCV	4	16	**20%**
CVC	9		
CCYV	0		
CYV	0		
CYVC	1		
CCVC	0		
CVCC	2		

3. English version of the detailed intervention procedure: phonological short-term memory training

SPEECH-LANGUAGE INTERVENTION PROCEDURE – STUDY 1

English version

Target : Phonological Short-Term Memory (pSTM)

1] Activities conducted during a typical session:

Core Activities

Activity 1 – Immediate Repetition of Meaningful Items (words)
Objective: Stimulate immediate phonological recall using real words.
Instruction: Present a list of meaningful items (real words) adapted to the participant's level. Ask the participant to repeat each item immediately after hearing it.
Format: Oral presentation; individual repetition; no feedback provided unless requested.

Activity 2 – Immediate Repetition of Non-Meaningful Items (pseudowords)
Objective: Reinforce phonological encoding and rehearsal through pseudowords.
Instruction: Present a list of non-meaningful items (pseudowords) adapted to the participant's level. Ask for immediate repetition.
Note: Ensure phonotactic plausibility and similar syllabic complexity across lists.

Activity 3 – Delayed Repetition of Meaningful Items (words)
Objective: Train short-term phonological retention with semantically rich material.
Instruction: Present the same list of meaningful items, but insert a brief distractor task (e.g., silent counting) before requesting repetition.

Activity 4 – Delayed Repetition of Non-Meaningful Items (pseudowords)
Objective: Maximize working memory load using delayed pseudoword repetition.
Instruction: Same structure as Activity 3, but with pseudowords.

Intermediate Task

Position in session: After Activity 2 / After Activity 4

Objective: To provide a brief motivational and attentional reset between demanding tasks.

Instruction: Present short, age-appropriate paragraphs on a slide. Whenever possible, select content related to the participant's personal interests. Invite the participant to read aloud and engage in a short conversation (approximately 5 minutes).

Notes: This activity is non-evaluative and aims to foster therapeutic alliance and attention maintenance.

Figure 1 : Structured sequence of tasks implemented in a standard intervention Session:

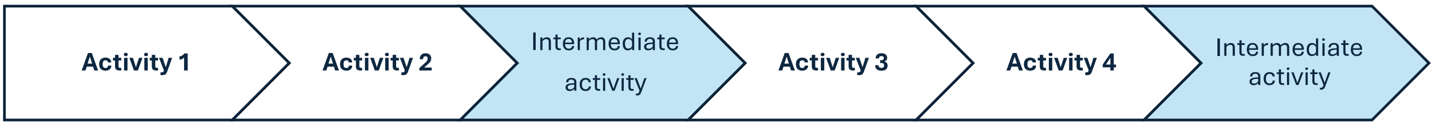

2] Important reminders for session preparation and documentation :

⚠ RANDOMIZE THE LISTS

Each slide set includes a pool of 25 target-level items (highlighted in pink) and 6 lower-level items (highlighted in yellow, except for the 2-syllable level). A total of 31 words will be presented to the participant in each session (25 for the 2-syllable level).
Before beginning the session, ensure that the randomization option is activated.
Procedure: Go to the "View" tab, then select "Macros" (on the far right of the ribbon). Click on "sort_rand", then "Run".

⚠ INSTRUCTION DELIVERY

Ensure that the task instructions are clearly understood by the participant. Maintain a supportive and respectful environment, and be attentive to any signs of discomfort or distress.
Do not hesitate to check in regularly regarding the participant's motivation and fatigue levels.

⚠ RECORDING THE SESSION

Be sure to record the session. At the beginning of the recording, ask for and confirm the participant's consent, in accordance with the guidelines outlined in the initial evaluation form (Phase 1 and Phase 2).
Save the video using the following format: participant code + session number (out of 15) + session date.
Example: IT1_2/15_280072023

⚠ DATA ENTRY

Individual Excel spreadsheets are available for each participant. At the end of each session, enter the total score obtained per target level and item. These values are essential for conducting repeated-measures analyses.
If preferred, scores may be recorded manually on paper during the session, provided that they are subsequently transferred to the Excel sheet.

⚠ SESSION LOG (FOLDER ENTRY)

Complete the session follow-up log, including observational notes (e.g., signs of fatigue, participant feedback, specific needs or concerns, etc.).

3] Instructions, Scoring, and Hierarchical Cueing Procedure

Instructions and hierarchical cueing procedures – Study 1

Initial Instructions per Activity

Activity 1 – Immediate Repetition of Real Words

"I'm going to say some real words. You'll need to repeat each one right after I say it, okay? I'll help

you if needed. Shall we start?"
→ [Say the first word from the randomized list.]
Activity 2 – Immediate Repetition of Pseudowords
"I'm going to say some made-up words. Don't worry, these are just invented words that don't mean anything. I'll still ask you to repeat them as best you can."
→ [Say the first pseudoword from the randomized list.]
Intermediate Activity 1 *(after Activity 2)*
"You've been very focused—thank you! If you're up for it, I'd like to suggest a short break activity. Remember, when we first met, I asked if there were topics or hobbies you enjoy. We'll use those as a starting point for a short reading activity. Does that sound okay?"
If yes, continue:
"Here is a short text I'd like you to read aloud. I'll ask you a few questions afterward."
Sample questions (adapted to the participant's interests and comprehension):
- *"What does this topic make you think of?"*
- *"Is this something that resonates with you?"*
- *"Why do you think this subject might be important to you?"*

! Adjust language based on fatigue, comprehension, and memory skills.

Activity 3 – Delayed Repetition of Real Words
"I'm going to say some real words. This time, I'll ask you to repeat each word five seconds later, okay? I'll let you know when to go by saying 'Go!' I'll help you if needed. Ready to begin?"
→ [Say the first word from the randomized list.]
Activity 4 – Delayed Repetition of Pseudowords
"Now I'll say some made-up words. You'll need to repeat them five seconds later, okay? I'll say 'Go!' to let you know when to repeat. I'll help you if needed. Let's get started."
→ [Say the first pseudoword from the randomized list.]
Intermediate Activity 2 *(after Activity 4)*
"You've been really focused—we're now near the end of the session. If you agree, I'd like to suggest a short reading activity, just like earlier. Remember the topics you told me you liked? We'll use one for this little task."

If yes, continue:
"Here's a short text I'd like you to read aloud. I'll ask a few questions afterward."
Sample questions (adapted to content and participant):
- *"What does this topic mean to you?"*
- *"Are you personally sensitive to this theme?"*
- *"Why do you think this is important for you, for example?"*

End of Session
- Thank and congratulate the participant for their effort and engagement.
- Remind them of the next session's date.
- At the end of each week, inform the participant that a progress assessment will be conducted. Mention that this session may take longer.

Mid-Week Evaluation Instructions
During mid-week evaluations, the administrator administers only the short List A and the corresponding List B for the target level practiced during the week.
If, during the week, two consecutive sessions showed ≥80% success and led to a level increase, only the most frequently practiced level should be assessed (e.g., Sessions 3 and 4 used 2-syllable items → evaluate level 2 at week's end, even if level 3 was introduced in Session 5).

Example Instruction:
"For this evaluation, I'll ask you to repeat some words again. It may take about 10 to 20 minutes. I

won't be able to help you during this part. Remember, it's simply to understand how your brain is processing the words."

Administer the following for the practiced level(s):

- Real words, immediate repetition: Short List A + List B
- Pseudowords, immediate repetition: Short List A + List B
- Real words, delayed repetition: Short List A + List B
- Pseudowords, delayed repetition: Short List A + List B

Scoring Guidelines

- Full point is awarded for each correct repetition of the item.
- No point is given if the repetition is incorrect, even if corrected after cueing.

Feedback and Motivation Support

- For each correct response, the administrator should provide positive reinforcement to maintain engagement.
  Example: *"Yes, great job!"*
  However, do not repeat the word (e.g., *"Yes, that's right, well done, it was 'pharmacist'"* should be avoided to prevent additional exposure to the target).

Response to Participant Failure – Hierarchical Cueing Procedure

General supportive prompt:

"Almost! Let me help you a bit, okay?"

Level I – Verbal Syllable Cueing

- Administrator segments the word into syllables orally (e.g., "phar-ma-cist").
- Ask the participant to repeat the word normally (not syllabified).
- If participant repeats with syllables, say:
  "Great. Now can you try to say the word normally?"

Level II – Verbal + Visual Cueing

- Administrator segments the word and uses a visual slide with colored spheres, one per syllable.
- After cueing, stop screen sharing and say:
  "Now can you say the word normally for me?"

Level III – Verbal + Sustained Visual Cueing

- Same as Level II, but keep the visual support visible.
- Use the cursor to guide the participant as they repeat, one syllable at a time.
- Then say:
  "Perfect. Let's try something harder. Can you now say the word normally?"

STOP

If the participant is unsuccessful at Level III:

☑ Discontinue the trial. Offer reassurance.
☑ Move to the next item.
☑ If the participant seems discouraged, pause and explain:
"It's completely normal to find this challenging. Think of it like training your memory—it takes time and energy. What matters most is practicing regularly, not being perfect right away."

Figure 2. Hierarchical Cueing Chain for Failed Trials – Levels 1 to 3

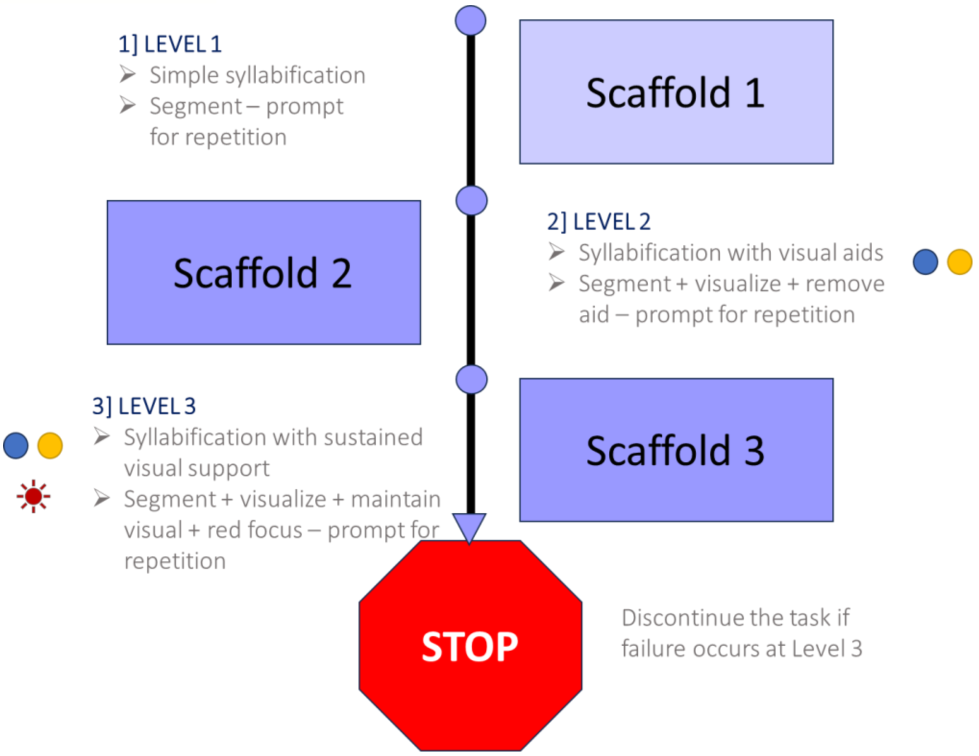
1] LEVEL 1
Simple syllabification
Segment – prompt for repetition
Scaffold 1
Scaffold 2
2] LEVEL 2
Syllabification with visual aids
Segment + visualize + remove aid – prompt for repetition
3] LEVEL 3
Syllabification with sustained visual support
Segment + visualize + maintain visual + red focus – prompt for repetition
Scaffold 3
STOP
Discontinue the task if failure occurs at Level 3

4. French version of the detailed intervention procedure: phonological short-term memory training

PROCÉDURE INTERVENTION ORTHOPHONIQUE ETUDE 1

Version française

Entraînement MPCT

1] Activités réalisées pendant une séance-type :

Activité 1 : Faire répéter directement la liste d'items signifiants (mots) correspondant au niveau du participant ;

Activité 2 : Faire répéter directement la liste d'items non-signifiants (pseudo-mots) correspondant au niveau du participant ;

Activité 3 : Faire répéter en différé la liste d'items signifiants (mots) correspondant au niveau du participant ;

Activité 4 : Faire répéter en différé la liste d'items non-signifiants (pseudo-mots) correspondant au niveau du participant.

Activité intermédiaire (entre activité 2 et 3 puis à la fin de l'activité 4) : Faire lire de courts paragraphes sur diapositive. Si possible en lien avec les goûts du participant et échanger sur le sujet (tâche intermédiaire de 5 minutes simplement pour motiver le participant et maintenir son attention).

Figure 1 : Enchaînement des activités à réaliser au cours d'une séance-type :

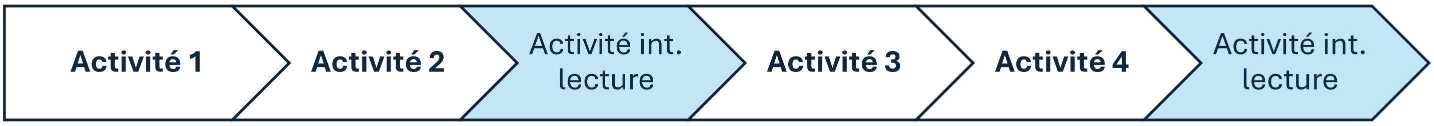

2] Points de vigilance :

! RANDOMISER LES LISTES : Un jeu de 25 items du niveau-cible (en **rose**) et de 6 items du niveau inférieur (en **jaune** sauf pour le niveau 2 syllabes) est intégré à des diapositives équipées d'une option de randomisation. 31 mots seront donc présentés au participant à chaque séance (25 mots pour le niveau 2 syllabes). Veiller à cliquer sur cette option avant de commencer la séance. **Procédure :** se rendre sur l'onglet « *Affichage* » puis option « *Macros* » (à droite du ruban) puis cliquer sur « *sort_rand* » puis « *exécuter* ».

! CONSIGNE : s'assurer que la consigne est bien comprise et veiller à maintenir un cadre bienveillant et une écoute suffisante pour repérer un moment d'inconfort pour le participant. Ne pas hésiter à l'interroger régulièrement sur sa motivation et sa fatigue.

! ENREGISTRER : veiller à enregistrer la séance et demander, au début de l'enregistrement, l'accord pour l'enregistrement (cf : règlementations et consignes dans le formulaire d'évaluation initiale phase 1 et phase 2). Veiller à enregistrer la vidéo en utilisant le code participant + le numéro de séance sur 15 + la date de la séance. e.g. IT1_2/15_280072023.

! COLLECTER : des fichiers excels individuels sont disponibles pour chaque participant pour intégrer la note totale obtenue à la fin de la séance. Ces totaux par niveaux-cibles et items serviront à réaliser nos mesures répétées. Il est possible de comptabiliser sur feuille tant que le report est ensuite fait sur le tableau excel.

! CARTABLE : remplir le fichier de suivi et indiquer des éléments observés (fatigue, perception du patient, besoins etc.).

3] Consignes, pondération et indiçage hiérarchisé :

Consignes et procédures d'indiçage hiérarchisé dans le cadre de l'étude 1 :

1] Consigne initiale Activité 1 mots immédiats : « Je vais vous dire des mots qu'il faudra répéter tout de suite après d'accord ? Je vous aiderai en cas de besoin, on commence ? [Dire le premier mot de liste randomisée] »

2] Consigne initiale Activité 2 pseudo-mots immédiats : « Je vais vous dire des mots qui n'existent pas. Je vous rassure ces mots ont été inventés et ne veulent rien dire. Je vous demande de les répéter malgré tout le mieux possible. [Dire le premier mot de liste randomisée] »

# Consigne activité intermédiaire 1 : « Vous avez été très attentive.tif. Je vous propose de faire une petite activité intermédiaire si vous le voulez bien. (Si c'est le cas ensuite :) Vous vous rappelez lorsque nous nous étions vus la première fois je vous avais demandé si vous aviez des passions ou des sujets qui vous intéressaient. Nous allons un peu en parler à travers une activité de lecture si vous le voulez bien. (Sinon, passer directement à :) Regardez, voici un petit texte que je vais vous demander de lire à voix haute. Je vais vous poser quelques questions dessus : exemples de questions (en lien avec le thème mais aussi les goûts du participant) « qu'est-ce que ce sujet vous inspire ? ; êtes-vous sensible à ce sujet ? Pourquoi c'est important pour vous par exemple que …. ? etc. » (ces questions sont à adapter/simplifier selon les capacités de compréhension, de rétention de l'information, de la fatigue du participant)

3] Consigne initiale Activité 3 mots différés : « Je vais vous dire des mots qu'il faudra répéter 5 secondes après d'accord ? Je vous donnerai le départ en disant « go ! » Je vous aiderai en cas de besoin, on commence ? [Dire le premier mot de liste randomisée] »

4] Consigne initiale Activité 4 pseudo-mots différés : « Je vais vous dire des mots qui n'existent pas qu'il faudra répéter 5 secondes après d'accord ? Je vous donnerai le départ en disant « go ! » Je vous aiderai en cas de besoin, on commence ? [Dire le premier mot de liste randomisée] »

# Consigne activité intermédiaire 2 : « Vous avez été très attentive.tif et nous arrivons à la fin de la séance. Je vous propose de faire une petite activité intermédiaire si vous le voulez bien. (Si c'est le cas ensuite :) Vous vous rappelez lorsque nous nous étions vus la première fois je vous avais

demandé si vous aviez des passions ou des sujets qui vous intéressaient. Nous allons un peu en parler à travers une activité de lecture si vous le voulez bien. (Sinon, passer directement à :) Regardez, voici un petit texte que je vais vous demander de lire à voix haute. Je vais vous poser quelques questions dessus. » exemples de questions (en lien avec le thème mais aussi les goûts du participant) « qu'est-ce que ce sujet vous inspire ? ; êtes-vous sensible à ce sujet ? Pourquoi c'est important pour vous par exemple que … ? etc. »

***5]* Fin de la séance :** remercier et féliciter le participant pour sa disponibilité et son endurance. Puis rappeler la date de la prochaine séance. À la fin de chaque semaine, la fin de séance diffère. Il est nécessaire de rappeler au participant que c'est le temps de l'évaluation des effets de l'intervention et qu'il va donc falloir rester un peu plus longtemps pour cela.

Consigne d'évaluation intermédiaire : L'administrateur fait passer uniquement la liste A courte et la liste B correspondant au niveau-cible travaillé pendant la semaine. Dans le cas où, au cours de la semaine, deux séances coup-sur-coup ont obtenu un score de 80% et plus de réussite et ont amené l'administrateur à passer au niveau supérieur, le niveau-cible majoritairement travaillé (2/3) doit être évalué (par exemple, séance 3, niveau-cible 2 syll = 80% ; séance 4, niveau-cible 2 syll = 90% ; séance 5 > passage au niveau-cible 3 syll. A la fin de la semaine, l'administrateur n'évalue que le niveau-cible majoritairement traité : niveau 2 syll). « Pour cette évaluation, je vais aussi vous demander de répéter des mots. Cela peut durer entre 10 et 20 minutes. Je ne pourrai pas vous aider. Rappelez-vous, c'est simplement pour comprendre comment fonctionne le traitement » puis reprendre les consignes pour chaque niveau-cible travaillé avec les listes correspondantes : mots immédiats Liste A courte + Liste B du niveau ciblé en séance, pseudo-mots immédiats Liste A courte + Liste B du niveau ciblé en séance, mots différés Liste A courte + Liste B du niveau ciblé en séance, pseudo-mots différés Liste A courte + Liste B du niveau ciblé en séance.

Pondération : Si le participant répète correctement (sans erreur) l'item, accorder le point. En cas d'erreur – même si répétition correcte après indiçage – le point n'est pas accordé.

En cas de réussite, l'administrateur veille à transmettre régulièrement des rétroactions positives afin de maintenir la motivation du participant sur ses productions : *« oui, bravo ! »* mais ne pas répéter le mot une nouvelle fois, type : « oui c'est ça, bravo ! c'est bien pharmacien » (pour éviter une nouvelle exposition de l'item)

Comportement à adopter en cas d'échec :

Consigne : « C'est presque ça. Je vais vous aider un peu d'accord ? »

I) Premier niveau d'indiçage : Syllabation simple de l'administrateur. L'administrateur va scander l'item échoué en le syllabant puis demander au participant de répéter le mot normalement (pas comme l'administrateur). Si le participant répète correctement et en syllabant, l'administrateur doit demander : « très bien est-ce que vous pouvez me dire le mot normalement maintenant ? »

II) Deuxième niveau d'indiçage (si niveau 1 échoué) : Syllabation de l'administrateur avec étayage visuel. L'administrateur va scander l'item échoué en le syllabant et en utilisant la diapositive d'aide qui symbolise visuellement chaque syllabe (des sphères de couleur). Une fois

terminé, l'administrateur enlève le partage d'écran puis demander au participant de répéter le mot normalement (pas comme l'administrateur). Si le participant répète correctement et en syllabant, l'administrateur doit demander : « très bien est-ce que vous pouvez me redire le mot normalement maintenant ? »

III) Troisième niveau d'indiçage (si niveau 2 échoué) : <u>Syllabation de l'administrateur avec étayage visuel laissé à disposition.</u> Pour cet indiçage, l'administrateur va comme pour le niveau 2, scander l'item échoué en le syllabant et en utilisant la diapositive d'aide qui symbolise visuellement chaque syllabe (des sphères de couleur). Une fois terminé, l'administrateur laisse le partage d'écran avec le bon nombre de sphères correspondantes et avec son pointeur demander au participant de répéter le mot en balayant avec lui chaque syllabe à répéter. Si le participant répète correctement et en syllabant, l'administrateur doit demander : « très bien c'est parfait. On va tenter plus loin : est-ce que vous pouvez me redire le mot normalement maintenant ? »

IV) Si arrivé au niveau 3, le participant n'arrive pas à produire le mot, arrêter, le réconforter et continuer vers l'item suivant. Si le participant se bute à cet échec, marquer une pause et rappeler que c'est un apprentissage pour « muscler la mémoire » et que nécessairement cela prend du temps et de l'énergie. L'important c'est d'être régulier plutôt que de performer tout de suite.

Figure 2 - Chaîne de traitement des étayages à proposer niveau après niveau (progressif) échec 1 à 3 :

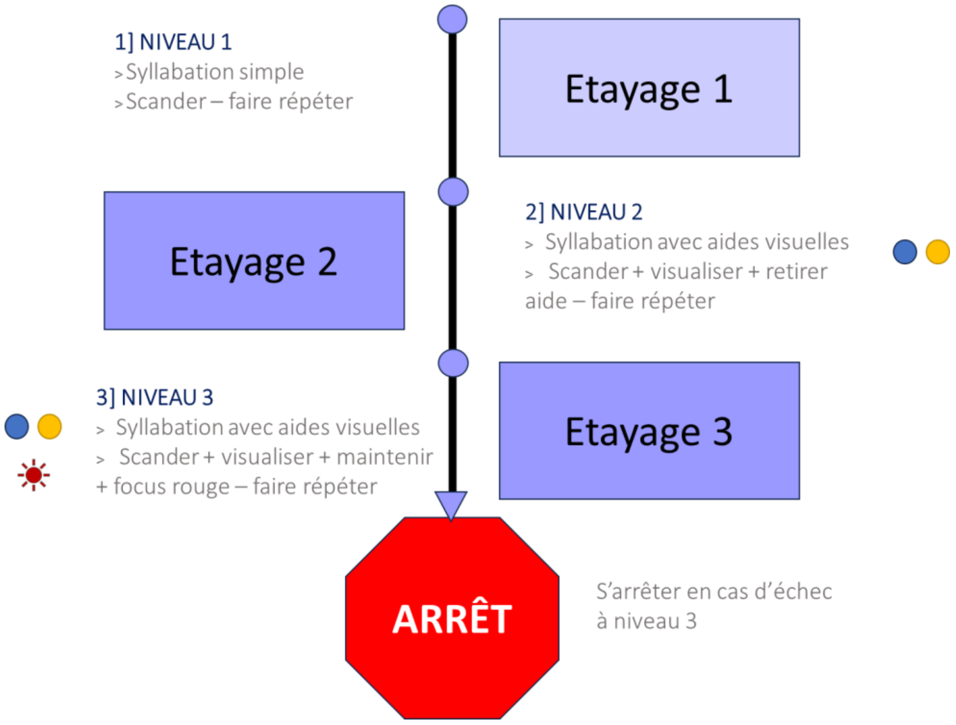

Supplement: Supplementary file 1 [file Data_Sheet_1.pdf]
